# The Pathway Is Clear but the Road Remains Unpaved: A Scoping Review of Implementation of Tools for Early Detection of Cerebral Palsy

**DOI:** 10.3390/children12070941

**Published:** 2025-07-17

**Authors:** Álvaro Hidalgo-Robles, Javier Merino-Andrés, Mareme Rose Samb Cisse, Manuel Pacheco-Molero, Irene León-Estrada, Mónica Gutiérrez-Ortega

**Affiliations:** 1 Facultad de Educación, Universidad Internacional de La Rioja, 26006 Logroño, Spain; alvaro.hidalgo@unir.net (Á.H.-R.); manuel.pacheco@unir.net (M.P.-M.); irene.leon@unir.net (I.L.-E.); 2 INAT (Early Intervention Research Team—Equipo de Investigación en Atención Temprana), Universidad Internacional de La Rioja, 26006 Logroño, Spain; 3 Toledo Physiotherapy Research Group (GIFTO), Faculty of Physiotherapy and Nursing, Universidad de Castilla-La Mancha, 45071 Toledo, Spain; javier.merino@uclm.es; 4 Toledo Physiotherapy Research Group (GIFTO), Instituto de Investigación Sanitaria de Castilla-La Mancha (IDISCAM), 45004 Toledo, Spain; 5 Independent Researcher, Sydney, NSW 2032, Australia; maremerose.samb@gmail.com; 6 Facultad de Educación y Trabajo Social, Universidad de Valladolid, 47011 Valladolid, Spain

**Keywords:** cerebral palsy, early detection, implementation science, General Movement Assessment, HINE, MRI, assessment tools, barriers and enablers

## Abstract

**Background/Objectives**: International guidelines recommend the combined use of the General Movement Assessment (GMA), Hammersmith Infant Neurological Examination (HINE), and magnetic resonance imaging (MRI) to support early and accurate diagnosis of cerebral palsy (CP). However, their implementation remains inconsistent. This study aimed to map their reported global use and identify associated enablers and barriers. **Methods**: A scoping review was conducted following JBI and PRISMA-ScR guidelines. Systematic searches were performed in PubMed, Cochrane, PEDro, ProQuest, Web of Science, and Scopus. Eligible studies were charted and thematically analyzed, focusing on tools use and implementation factors at individual, organizational, and system levels. **Results**: Fourteen articles (seven surveys, seven implementation studies) from seven countries met the inclusion criteria. While awareness of GMA, HINE, and MRI was generally high, routine clinical use was limited—particularly outside structured implementation initiatives. Major barriers emerged at the system level (e.g., limited training access, time constraints, lack of standardized referral pathways) and social level (e.g., unclear leadership and coordination). **Conclusions**: The limited integration of GMA, HINE, and MRI into routine practice reflects a persistent “know–do” gap in early CP detection. Since implementation is shaped by the dynamic interplay of capability, opportunity, and motivation, bridging this gap demands sustained and equitable action—by addressing system-wide barriers, supporting professional development, and embedding early detection within national care pathways.

## 1. Introduction

Early and accurate diagnosis of cerebral palsy (CP) is an urgent public health priority, consistently underscored by families who report that it should occur earlier and without the emotional burden of a late diagnosis [1,2,3]. The period before 6 months corrected age is considered a critical window in early development, when the brain shows heightened plasticity and is especially responsive to interventions that promote motor learning and functional reorganization [4,5]. Identifying infants at “high-risk” for CP during this phase opens the door to timely evidence-based interventions, optimizing outcomes and supporting families [6,7]. However, access to such interventions depends on the timely and accurate identification of infants at risk of CP.

In response to this need, international clinical guidelines published in 2017 synthesized the best available evidence and proposed a structured diagnostic framework for infants at “high-risk” [8]. These guidelines recommend combining the General Movements Assessment (GMA) [9], Hammersmith Infant Neurological Examination (HINE) [10], and magnetic resonance imaging (MRI). Together, the assessment of spontaneous movement patterns, neuromotor examination, and brain imaging provides a comprehensive view of the functional and structural integrity of the nervous system that maximizes diagnostic accuracy and enables detection from as early as 3 months [11]. Importantly, guidelines aim to reduce the heterogeneity of diagnostic pathways observed in clinical practice [12] and to ensure the consistent application of evidence-based diagnostic approaches across diverse healthcare contexts.

Despite this global consensus, integration of GMA, HINE, and MRI into routine practice has been inconsistent [13], and early diagnosis remains a significant challenge [14]. The literature has identified multiple barriers—e.g., limited training in recommended tools, lack of organizational readiness, or insufficient alignment with evidence-based care pathways—that impact the widespread adoption of these tools across countries, particularly in low- and middle-income contexts [13,15]. This challenge reflects broader, well-documented patterns in healthcare, where the implementation of clinical guidelines is often unpredictable, slow, and highly context-dependent [16].

Knowledge-to-practice gaps in early detection of CP have significant consequences, including delayed diagnoses, missed opportunities to deliver evidence-based interventions highlighted by international clinical guidelines [17], and inequitable access to services. Addressing potential barriers requires structured approaches, where implementation science, knowledge translation, and successful international experiences become essential [18,19].

The objective of this scoping review is to map the current global evidence on the implementation of recommended tools for early detection of CP. Specifically, this review aims to (1) identify the reported frequency of use of recommended tools; (2) identify reported facilitators and barriers to implementation; and (3) inform future implementation efforts by highlighting knowledge gaps.

## 2. Materials and Methods

This scoping review was conducted in accordance with the Joanna Briggs Institute (JBI) methodological guidelines for scoping reviews [20]. Reporting follows the Preferred Reporting Items for Systematic Reviews and Meta-Analyses Extension for Scoping Reviews (PRISMA-ScR) (Supplementary Table S1) [21]. The protocol was prospectively registered on the Open Science Framework on 14 May 2025 (http://osf.io/jwdvm).

The review was designed to address the following research questions:

1. What is the reported frequency of use of GMA, HINE, and MRI in the early detection of CP across countries and healthcare systems?
2. What contextual enablers and barriers influence the implementation of these tools in clinical practice?

### 2.1. Search Strategy

A systematic literature search was conducted up to May 2025 across the following databases: PubMed, Cochrane Library, PEDro, ProQuest, Web of Science, and Scopus. Full details of the electronic search strategy are provided in Supplementary Table S2.

To capture additional relevant evidence, the search was supplemented by targeted hand-searching of the reference lists from included articles and grey literature sources [21]—e.g., preprints, doctoral theses, conference abstracts, and government/institution reports. Additionally, Google Scholar citation tracking (descendancy approach) was used to identify recent studies citing any of the included articles.

### 2.2. Inclusion and Exclusion Criteria

Eligibility criteria were defined using the Participant–Concept–Context (PCC) framework:

- Participants: Studies involving healthcare professionals, clinical teams, or healthcare systems engaged in early detection of CP.
- Concept: Studies investigating the use, implementation, or integration of recommended early detection tools for CP—GMA, HINE, neuroimaging, and other tools cited in the international guidelines. This included research describing awareness, frequency of use, barriers and facilitators to implementation, or contextual factors influencing clinical practice.
- Context: Studies conducted in any healthcare or service setting, across all geographic and economic contexts (high-, middle-, and low-income countries), including clinical, community-based, and public or private health settings.

Eligible evidence included quantitative (e.g., observational, cross-sectional, survey), qualitative, and mixed-methods studies. Grey literature was included to enhance the comprehensiveness of the evidence capture, particularly given that CP detection implementation efforts are often driven by institutions that may report findings through internal documents, conference proceedings, or unpublished surveys. These sources were interpreted with caution—for example, preprints were clearly identified and their results contextualized accordingly within the synthesis. No restrictions were applied regarding publication language or date.

Studies were excluded if they focused solely on diagnostic accuracy, psychometric properties, or implementation strategies without providing data on the practical use or frequency of early detection tools in clinical contexts.

### 2.3. Screening Process

Database research was conducted by one author (Á.H.-R.), supplemented by hand-searching of reference lists and secondary searches. Two reviewers (Á.H.-R. and M.R.S.C.) independently screened titles and abstracts for eligibility. Any disagreements at either stage were resolved through discussion and consensus with a third reviewer (J.M.-A.). Full-text reviews and inclusion/exclusion decisions were also conducted independently by the same authors, with rationales for decisions documented and agreed upon through discussion.

### 2.4. Data Extraction, Synthesis, and Appraisal

For the extraction of variables from the included articles, two reviewers (Á.H.-R. and J.M.-A.) independently extracted data including country, method of data collection (e.g., survey, focus groups), population size (*n*), healthcare setting, provider background and experience, use of recommended tools, use of alternative tools, and reported enablers and barriers to implementation. Any disagreements during data extraction were resolved through discussion and consensus between the two reviewers, with a third reviewer (M.R.S.C.) consulted if needed.

Quantitative outcomes—such as reported frequencies of tools use—were tabulated and used to describe the implementation status across different settings and countries. Qualitative data were analyzed thematically, based on textual content from open-ended survey responses, focus groups, and qualitative interviews. Quotes and themes were extracted independently by two reviewers and grouped into preliminary categories. These categories were refined through discussion and mapped onto implementation constructs, labeled according to domains commonly cited in the literature (e.g., system-level or social-level factors). The number of respondents supporting each theme was extracted as reported by the primary authors, without recoding or reinterpreting their classifications. Quantitative and qualitative findings were integrated in a narrative synthesis to explore patterns of convergence and divergence across study types and contexts.

## 3. Results

**Figure 1:**
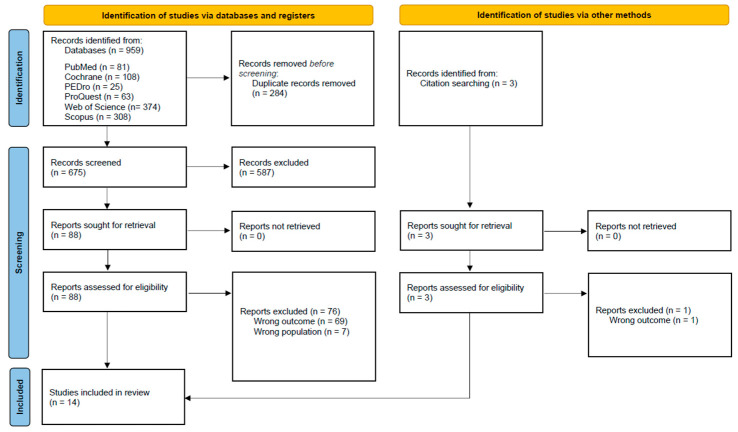
PRISMA 2020 flow diagram [22].

The database search yielded a total of 959 records, as shown in the PRISMA 2020 flow diagram (Figure 1). After removing duplicates, 675 unique records remained. Titles and abstracts screening reduced the selection to 91 articles—including 3 identified through hand-searching. Following independent full-text review and consensus discussion, 77 articles were excluded due to (a) wrong outcome (*n* = 70) or (b) wrong population (*n* = 7) (Supplementary Table S3).

### 3.1. Geographic and Professional Overview

This review includes seven survey-based studies conducted in the United States (US) [23], New Zealand [13], Germany [24], Spain [25], Brazil [26], the UK (England, Scotland, and Wales) [27] (preprint), and the US states of Maryland and Delaware [28]. In parallel, seven implementation studies from the US [29,30,31,32], Australia [33], New Zealand [34], and Spain [35] evaluated the adoption of individual tools or international guidelines, reporting pre- and/or post-implementation metrics. Characteristics of included articles are summarized in Appendix A.

Participants were primarily physical therapists, occupational therapists, and medical providers. In this context, 8 out the 14 studies [13,24,27,28,30,31,32,34] included both providers who reported providing a diagnosis of CP and those involved in the assessment of infants at risk, even if not formally responsible for diagnosis. Sample sizes ranged from 11 [35] to 269 [23].

### 3.2. Evidence (Research, Clinical Experience, and Families) and Awareness of Early CP Detection and Diagnosis

In New Zealand, 75% of providers acknowledged the usefulness of research evidence and its alignment with their clinical understanding, while 63% agreed that the key messages for implementing best-practice recommendations were clear [34]. Awareness of the feasibility of early CP diagnosis has grown in this context, supported by increased visibility and targeted awareness-building efforts—such as the Early Identification and Intervention for Infants Network (Ei3) initiative [32]. This program developed resources and deliverables for caregivers, providers, and policymakers, together with a framework for dissemination. As a second step, capacity-building efforts included HINE training, both basic and advanced Prechtl’s GMA training, scholarship provision, stakeholder feedback mechanisms, and an implementation conference.

In pre/post-HINE-training evaluation (mean *n* = 27), significant improvements were observed in providers’ ability to identify CP (*p* = 0.001), knowledge of early detection (*p* < 0.001), and ability (*p* < 0.001) and knowledge (*p* = 0.004) to implement international guidelines. A post-training survey also demonstrated increased understanding of the rationale for early detection of CP, their perceived role in early detection, and confidence in performing HINE [32]. Similarly, a pre/post-implementation study in Australia reported significant improvement in providers’ awareness that CP can be diagnosed early, shifting from “very little” to “very much” among 26 participants (*p* < 0.001) [30]. Most participants across studies agreed that CP can be diagnosed before 6 months of corrected age (e.g., 64% in Brazil [26]) or before 12 months (88% in Maryland and Delaware [28]).

However, despite widespread awareness, implementation remains limited in practice. In Maryland and Delaware, only 19% reported that children in their care received a CP diagnosis before 12 months of corrected age. Similarly, in Spain, physical therapists reported an average diagnostic age of 12.6, although 61% indicated that they typically referred children before their first year of corrected age. In Maryland and Delaware, 15 free-text responses explicitly suggested that health providers considered CP diagnosis to be delayed or late [28]. This perception of delay appears to be linked with low perceived consensus regarding the value of research evidence in early CP diagnosis: only 30% of providers in Auckland reported a clear consensus on its relevance [34].

Over 65% of providers in Auckland agreed that clinical experience was useful and an important source of knowledge, with 58% specifically considering their own experience as additional evidence. Nonetheless, opportunities for consensus and collaborative learning beyond the clinical workplace were reported to be limited [34].

Although 91% of providers valued family experience as a relevant source of evidence that helped shape their understanding of early CP detection and intervention, only 35% perceived consensus on its value, and just 18% reported routinely collecting family experiences in practice [34].

### 3.3. Referral Pathways for CP Diagnosis

Referral pathways for CP diagnosis and follow-up were often reported as unclear or non-standardized. In Spain, only 34% of the physical therapists indicated having a clear referral protocol when identifying a child at risk of CP [25], a figure that dropped to 14% in Maryland and Delaware [28]. Nearly half of the participants in these contexts stated that their workplace lacked any formal guidelines or referral procedures [28].

Where protocols existed, referral pathways varied widely and frequently lacked standardization. Referrals could involve a single health provider or a combination of two or three specialists [13]. The most commonly reported referral destinations included pediatric neurology (52% in Maryland and Delaware, 38% in Spain, 18% in New Zealand), general pediatrics (28% in Maryland and Delaware, 25% in Spain, 32% in New Zealand), and developmental pediatrics (48% in Maryland and Delaware, 15% in Spain, 34% in New Zealand). Additional specialists such as those in child developmental services, physical medicine and rehabilitation, or pediatric orthopedics were also identified [13,28].

Implementation initiatives—even those focused on individual tools like HINE—showed potential for improving organizational procedures. In one study, the proportion of providers responding affirmatively to the question “Does your workplace have any protocol, procedure or standardized reference guidelines for the referral of children at high risk of CP?” increased from 27% before training to 63% post-training [35].

### 3.4. Use of GMA, HINE, and MRI

**Figure 2:**
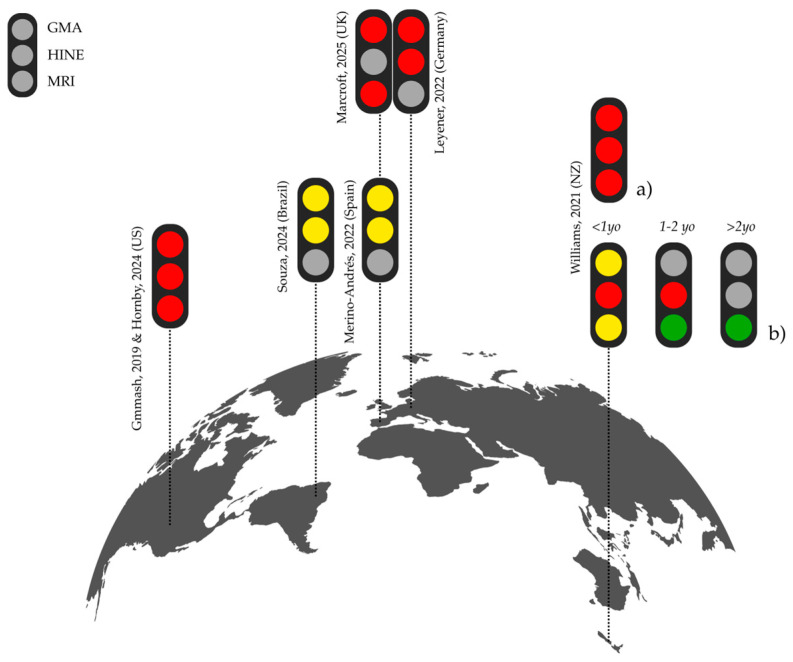
Global implementation of GMA, HINE, and MRI based on reported use in included studies [13,23,24,25,26,27,28] (individual provider and neonatal units reports). Color codes indicate reported level of tool use: red 🔴 (<25%), yellow 🟡 (25–50%, inclusive), green 🟢 (>50%), or gray (no data available, assessment not applicable to the age range or the age group participants work with). For data from Williams, 2021 [13], traffic lights panel show (**a**) professionals involved in providing diagnosis; (**b**) professionals not directly responsible for diagnosis.

Given the heterogeneity in how GMA, HINE, and MRI metrics were reported, these findings are best interpreted as a descriptive mapping of implementation patterns rather than as directly comparable quantitative rates. Figure 2 provides an overview of international surveys assessing these patterns [13,23,24,25,26,27,28].

Table 1, Table 2 and Table 3 summarize the reported use of recommended (strong or conditional) and alternative assessment tools for early detection of CP, categorized by data source: individual provider reports (Table 1), institutional/neonatal units (Table 2), and patient-level clinical records (Table 3).

#### 3.4.1. GMA

The diagnostic value of GMA was strongly supported, with 94% of providers endorsing its utility for early CP detection [34]. In terms of feasibility, 47% of participants considered it feasible to film GMA videos for all at-risk infants at the recommended timepoints (32–35 weeks, term, and 12–14 weeks), and 53% considered it feasible to have trained assessors available to review and interpret the recordings [34].

To these moderate feasibility perceptions was added a consistently low and variable use of GMA across countries and settings. Although 70% of providers in New Zealand reported being aware of GMA, nearly half of those responsible for diagnosis never used it, and this figure increased to 74% among providers not responsible for delivering the diagnosis [13]. In Brazil, 63% of providers were familiar with the tool, but only 27% used it in practice, and fewer than 20% applied it in combination with HINE [26]. In Spain, 41% of physical therapists knew about GMA, with 26% using it regularly for early detection [25]. In Germany, 87% of NICU providers were familiar with GMA—mainly through professional exchanges, the literature, and lectures—yet only 24% used it routinely [24], a percentage comparable to the 22.1% (*n* = 32) of the UK units surveyed [27]. Among German users, 44% had completed the Prechtl GMA Basic Training Course, 17% the Advanced Training Course, while 54% had no formal qualification. The tool was mostly administered by medical doctors (44%) and physical therapists (41%). In the United States, use was minimal: only 4% of participants in the national survey and 6% in Maryland and Delaware reported always using GMA [23,28].

Implementation efforts, however, led to significant improvements. One study reported an increase in GMA use from 17% during the pre-implementation phase to 61% after implementation (*p* = 0.041) [33]. Similarly, US data showed an approximate 50% increase in GMA when comparing the periods 2010–2017 and 2018–2022, following the publication of international guidelines [31].

Main barriers to GMA use included lack of training or uncertainty about how to administer and/or score the tool (*n* = 123), unfamiliarity with the tool (*n* = 20), lack of workplace support (*n* = 18), reliance on clinical signs and symptoms (*n* = 10), perception that it was outside the provider’s scope of practice (*n* = 14), and limited resources (*n* = 19) [13,25,28].

#### 3.4.2. HINE

The value of HINE was consistently recognized across studies. In New Zealand, 83% of providers endorsed its utility in the early diagnosis of CP [34]. Awareness of the tool ranged from 77% in New Zealand to 66% in Brazil and 41% in Spain [13,25,26]. Most providers in New Zealand (72%) considered it feasible to assess all at-risk infants at 12–14 weeks [34]. However, knowledge of HINE optimality scoring was limited—only 40% of participants in New Zealand and 11% in Brazil reported being familiar with it.

Despite these favorable perceptions, routine use of HINE was limited across most contexts. In New Zealand, 48% of providers diagnosing CP reported never using HINE for infants under one year, and 57% did not use it between ages one and two [13]. In Brazil, 37% used it in clinical practice [26], while in Spain, just 28% reported applying it with infants at risk of CP [25]. Use was even lower in the UK and US: 82% of the neonatal units in England, Scotland, and Wales did not use HINE, 70% of providers in Maryland and Delaware had never used it, and in a national US survey, <1% of participants reported using it to assess motor severity.

Notable progress in HINE usage was observed following targeted training programs. In both in the US [29]—where documentation increased from 37% to 90%—and Spain [35], specific training and implementation led to improvements. Approximately 17% of infants were assessed with HINE during the 2018–2022 period—following the publication of international guidelines—compared to non-use (0%) before 2018 [31].

In this sense, after training, providers increasingly “strongly agreed” that HINE supported referral processes, facilitated communication with medical teams and families, and could be successfully integrated into routine practice [35]. Training also enhanced providers’ confidence in the ability to use HINE in clinical practice [32]. Inter-rater reliability one year post-training showed excellent agreement—intraclass correlation coefficient of 0.99—for the total score [35]. Additional improvements were also observed in broader implementation studies: the percentage of HINE assessments performed ≤5 months increased by 27% and >5 months by 21% among infants referred under 5 months. Furthermore, for 76% of infants eligible for HINE (≤2 years old), recorded use increased from 0% to 5% [33].

The most frequently cited barriers to HINE use included lack of training or knowledge of how to administer and/or score the tool (*n* = 114), unfamiliarity with the tool (*n* = 26), lack of workplace support (*n* = 12), reliance on clinical signs and symptoms (*n* = 12), and limited resources (*n* = 12) [13,25,28]. Maitre et al. [29] further categorized pre-implementation challenges (*n* = 12) into three categories: (a) concerns about increased time demands related to documentation; (b) inconsistent provider knowledge due to professional diversity and lack of shared language; and (c) concerns that the neurological examination might disrupt workflow without providing tangible clinical benefit. Three weeks after the training, most providers described HINE as brief and easily integrated into routine care, though concerns remained regarding documentation within electronic medical records.

#### 3.4.3. MRI

MRI was the most widely recognized tool across studies, particularly valued for its role in early CP diagnosis. In New Zealand, 83% of providers considered MRI a valuable tool [34], with reported familiarity reaching 94% [13]. Similarly, 66% of therapists in Brazil reported being familiar with MRI [26]. In New Zealand, 72% of providers considered MRI justified for all term babies within the early detection pathway, and 56% considered its use feasible in routine practice [34].

While these figures suggest a strong recognition of MRI, evidence on its actual implementation following the introduction of guidelines remains mixed. In Australia, MRI use in infants ≤ 5 months increased markedly—from 33% (*n* = 2) to 77% (*n* = 161) [33]. In another longitudinal analysis, MRI was the only tool that maintained high use rates (~90%) when comparing data from 2018 to 2022 with 2010 to 2017 [31]

Its use in clinical practice appears to be constrained by barriers related to access, cost, and professional scope-of-practice. In New Zealand, although 81% of clinicians diagnosing CP viewed MRI as a recommended tool for infants under one year, nearly half reported never used it routinely, often citing it as outside their scope of practice (*n* = 7) [13]. In Maryland and Delaware, 70% of professionals had never used or referred for MRI [28]. In Spain, MRI was commonly requested but usually in combination with clinical history rather than GMA or HINE [25]. In Brazil, while MRI use by therapists was not specifically analyzed, referrals based on neuroimaging findings accounted for just 1% of cases, suggesting limited integration in early detection at the community level [26].

**Figure 3:**
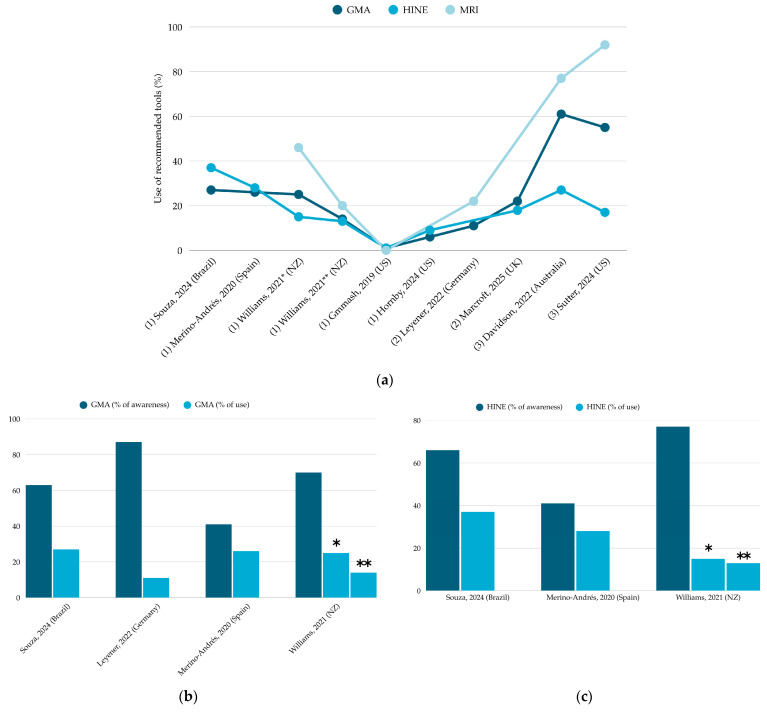
Graphical summaries of tool use: (**a**) Across all included studies [13,23,24,25,26,27,28,31,33], showing (1) individual provider report, (2) neonatal units report, and (3) patient-level data; (**b**) Comparing awareness versus use for GMA [13,24,25,26]; (**c**) Comparing awareness versus use for HINE [13,25,26]. For Williams et al. (2021) [13], the data are further stratified to distinguish professionals directly involved in diagnosis * from those who are not **.

Figure 3 illustrates patterns of use and awareness of GMA, HINE, and MRI across the included studies.

### 3.5. Use of Other Motor Assessments Tools with Strong or Conditional Recommendation

In addition to GMA, HINE, and MRI, several other tools have been identified in international guidelines as useful for early detection of CP, receiving either strong or conditional recommendations, depending on the strength of psychometric evidence in at-risk populations [8].

The Alberta Infant Motor Scale (AIMS) emerged as the most frequently reported. Its use was particularly high in Brazil (62%) and Spain (41%) but considerably low in the UK—where only 17% of surveyed neonatal services reported using it—and Maryland and Delaware (9%). Longitudinal data from the US indicate a decrease of ~10% in AIMS use between 2010 and 2017 and 2018 and 2022, falling to around ~30% [31].

In contrast, the Developmental Assessment of Young Children (DAYC) showed a different trend, with reported use in New Zealand ranging from 35 to 37%, and 16% among providers not directly responsible for diagnosis [13]. In the US, reported use ranged from approximately 5% to 12% and increased significantly in Maryland and Delaware, reaching 59% [23,28,31].

With the exception of Brazil (25%) [26] and the Waisman Center Newborn Follow Up Clinic in the US—where use reached ~50% during the 2018–2022 period [31]—use of the Test of Infant Motor Performance (TIMP) remained consistently low (<10%) across other countries. Use of the Motor Assessment of Infants (MAI) and Neuro-Sensory Motor Developmental Assessment (NSMDA) was generally infrequent in all contexts.

Interestingly, targeted implementation of individual tools (e.g., HINE) may contribute to increased familiarity with other recommended assessments such as GMA and AIMS. Nevertheless, this pattern was not consistent across all tools; in the case of TIMP, no increase in familiarity was observed following implementation [35].

### 3.6. Use of Alternative Assessment Tools

There was widespread reliance on clinical signs, medical history, and clinical experience or judgement [27]—particularly in contexts where standardized tools or formal referral pathways were unavailable or inconsistently implemented.

The Bayley Scales of Infant and Toddler Development were routinely used in the UK, Spain, and New Zealand—especially for children aged 1–2 years and older in the latter—often followed by tools such as HINE or DAYC [13,25,27]. The Peabody Developmental Motor Scales (PDMS-2) was widely used in the US [23,28]. Additional tools reported included the Dubowitz Neurological Assessment, Touwen Neurological Examination, and Ages and Stages Questionnaires (ASQ), particularly in pediatric primary care and neonatal follow-up programs.

In Brazil, nearly half of the therapists reported using neurodevelopmental approaches such as Bobath, which are primarily aligned with intervention philosophies rather than diagnostic accuracy. In Spain, the widespread use of the Vojta method—which includes an embedded assessment rationale—illustrates that more than half of the providers incorporate Vojta-based assessments into their clinical practice [25].

### 3.7. Enablers and Barriers

Facilitators and barriers to the use of early detection tools were explored, including both quantitative and qualitative data where available (Table 4). These factors were categorized into four interrelated domains: system-level, social-level, individual knowledge and perceptions, and clinical considerations.

1. **System-level factors** were widely reported across studies, with most barriers (*n* = 190) related to staffing constraints, time allocation, financial resources, and/or lack of referral pathways.
  Staffing and workload issues were a central concern. Providers described high caseloads and limited personnel, making it difficult to integrate new assessments into routine care, even when trained. A clinician in New Zealand summarized this as *“constantly in crisis mode and little time to prep for sessions or implement new tools”* [13]. This was echoed in Spain [25], where *“inflexible schedules”* led providers to rely on clinical judgement over standardized tools.
  Funding limitations were also frequently cited. Spanish physical therapists reported *“economic difficulties to access training”*, while similar concerns arose in contexts like New Zealand [13] and Maryland or Delaware [28], where a provider mentioned costs of training, materials, and time required for courses [13,28].
  Infrastructure and operational limitations further constrained implementation. In Auckland, fewer than half considered that resources—e.g., information technology (10%), financial resources (20%), and human resources (15%)—were adequate to support new practices. One participant observed: *“I do not see currently… that we have any of the resources (human and non-human) that are required for the implementation of this pathway […]”* [34].
  Delays in referral and the lack of standardized pathways compounded these issues. A provider in New Zealand highlighted the need for *“[…] clear guidelines on when and how to screen and refer”* [13], concerns echoed in Spain and the US, where bureaucratic hurdles and frequent protocol changes slowed adoption [25,28]. In contrast, integrated follow-up programs or established referral frameworks were viewed as critical enablers, facilitating joint assessments and smoother coordination: *“This makes referral, joint assessment and collaboration much easier for families and for us”* [13].
  Additional system-level enablers (*n* = 49) included organizational support and protected time for training, as reported in Spain, Maryland, and Delaware [25,28]. In this context, a provider in New Zealand highlighted the need for broader access to education opportunities: *“Whilst I have read about HINE and GMA, haven’t attended training on GMA—if this training could be made more available and accessible to therapists in NZ that would be amazing”*.
2. **Social-level** enablers (*n* = 89) and barriers (*n* = 73), though less frequently reported than system-level barriers, played a critical role. Leadership and administrative support, peer collaboration, and organizational culture shaped implementation outcomes.
  Leadership consistently emerged as a key driver of success, with 65% of participants in New Zealand feeling they had the power and authority to influence implementation. Support from supervisors and administrators keen to follow evidence-based practice was identified as a facilitator [13,28]. However, several participants emphasized the challenge of limited leadership involvement: *“Staff not wanting to change the status quo from how it’s always been done”* [13]. Findings from Mulqueeney et al. (2024) [34] further illustrated this tension: only half of participants felt clear on their implementation roles, and less than a third had been involved in planning. Focus groups described leadership both as a barrier and an enabler. One participant observed it worked best when *“a really passionate person at the top [was] talking about brain care all the time”* [34]. Equity also emerged as a leadership concern: *“If we don’t make an effort to make this whole thing equitable then it won’t be”* [34].
  Multidisciplinary collaboration and peer support were frequently reported as critical enablers operating at different levels. Providers described informal peer exchanges and shared experiences [13,25], alongside the need for structured collective action and operational alignment between teams (*“Would need the team to work together to change practice—would need an algorithm and help with scheduling”* [28]). Peer review and supervision reinforced good practice [25] In New Zealand. Survey findings supported this collaborative culture: most team members were open to change—with only 19% preferring to maintain current practices—and between 52% and 70% agreed that their organization valued open communication and dialogue, relationships, and teamwork [34].
  In contrast, lack of coordination across teams and limited indirect service time were identified as barriers [25]. As one Spanish provider summarized: *“A change of mentality is needed”*—toward fostering a culture of shared leadership, interprofessional collaboration, and peer-driven support.
3. **Health professional knowledge and perceptions** consistently emerged as key factors of implementation—more like enablers (*n* = 80) than barriers (*n* = 57). In New Zealand, 60% of participants believed they had the skills and knowledge to implement the recommendations [34]. Access to professional development and knowledge sharing—e.g., through conferences, external courses attendance, or *“use of different apps, social networks and being connect to the field of pediatrics”* [25]—facilitated implementation. *“Guidelines’ development”* [25] and clinical pathways were also described as both relevant facilitators and barriers—and not just their absence in the workplace, but, for example, *“the many steps one needs to go through to be OKed to use a specific assessment tool”* [28].
  Importantly, both “clinical experience” and “family experience” were commonly cited as forms of evidence guiding decision-making, although with mixed consensus [34]. While most New Zealand providers valued family experience, there were 61 quotes identifying family experience as a barrier and 35 as an enabler, highlighting tensions around low knowledge and/or engagement (*“Sharing information is the keystone to having parents integrated in care”* [34] or *“Lack of families’ involvement”* [25]), myths, taboos, and misconceptions about CP that could complicate early detection discussions. Some expressed concern about causing undue stress in families who ultimately did not receive a CP diagnosis, describing this as potentially “paternalistic” [34].
  Professional perceptions of early diagnosis also intersected with broader cultural attitudes. There was no current consensus amongst doctors around the need/importance of early diagnosis of CP, feeling uncomfortable with giving it early and the avoidance of tough conversations (*“Severe is fine, I’m very happy to make the call. But under six months, you know, unless they’re severe, then, I start to talk about, well, risk of or, you know, maybe or needing more information. Very uncomfortable around making a definite diagnosis at that stage”*), or reliance on tools (*“While the sensitivity and the specificity of the MRI and the HINE are high, their positive predictive value is not. So… there’s reasonable chance that if you say someone has CP they don’t actually”*) [34]. Others, however, viewed early and transparent communication—including the use of family experience and clinical intuition—as key to building trust, tailoring care, and initiating appropriate intervention.
4. **Clinical considerations and individual drive**, though less frequently reported (*n* = 19 enablers; *n* = 17 barriers), shaped decisions around tools use in meaningful ways. Providers often described self-driven motivation as a critical enabler for implementation efforts [13].
  In contrast, increasing case complexity was reported to reduce the capacity for closely monitoring infants at risk [13]. Others highlighted variability in clinical routines (*“I have different patient groups”* [25]). Finally, some providers expressed concern that complex or time-consuming assessment could disrupt the flow of clinical care without delivering tangible benefits to patients [29].

## 4. Discussion

Early detection of CP is strongly supported by international, evidence-based clinical guidelines [8], which advocate for the systematic use of standardized tools to enable timely and accurate diagnosis. This scoping review maps the reported global frequency of use of these recommended tools among healthcare providers, highlighting a range of contextual factors—both enablers and barriers—that influence their implementation across Australia, Brazil, Germany, New Zealand, Spain, the UK, and the US [13,23,24,25,26,27,28,29,30,31,33,34,35].

Despite robust evidence supporting GMA, HINE, and MRI as highly predictive tools for early CP detection before five months corrected age—with reported accuracy levels of up to 97% [11]—and their proven effectiveness in lowering the age of CP diagnosis in high-risk infant follow-up settings [19], their routine clinical implementation globally remains limited. Reported use of GMA ranged from as low as 4 to 6% in the US [23,28] to 11 to 27% in Germany, New Zealand, Spain, Brazil, and the UK [13,24,25,26,27]. Similar disparities were observed with HINE, where regular use was reported by 28–37% of providers in Spain and Brazil and by <1% in a national US survey [23]. MRI, while more widely recognized, was still inconsistently used—with up to half of surveyed providers reporting non-use or non-referral [13]. However, in settings where early detection programs had been introduced, GMA usage increased to 55–61% [31,33], and HINE documentation rates improved dramatically (up to 90%) with targeted training initiatives [29].

These findings underscore a key implementation gap: the tools are known, the evidence is strong, and many professionals are motivated—yet, their use is still hindered by a range of structural, organizational, and contextual barriers. Our synthesis supports the view that moving from knowledge to action requires more than individual training; it demands context-sensitive, coordinated strategies that align clinical knowledge, coordinated pathways, institutional commitment, and supportive policy frameworks.

Building on implementation science is essential to address what implementation science has long described as the “know–do gap”—a persistent disconnect between knowledge gained and real-world practice. Surveys show that health providers are often not unaware or unwilling—they are unsupported and limited by insufficient time, workload, staffing, and funding [13,25,28,34].

Frameworks such as Integrated Knowledge Translation and Action [33], Knowledge-to-Action cycle [36], and Promoting Action on Research Implementation in Health Services [34], together with a growing body of implementation literature, point to diverse strategies that can improve adoption of evidence-based practices in early CP detection. Applying the COM-B model [37] and the Consolidated Framework for Implementation Research (CFIR) [38] clarifies both the nature of the behavioral changes needed—capability, opportunity, and motivation—and the multi-level factors that influence successful implementation and sustainment of early detection practices.

- Building Capacity… And Then What?

Limited proficiency and confidence in applying early detection tools can be effectively addressed through structured training programs in GMA and/or HINE, a need consistently identified by providers across contexts [13,25]. Given the historically ad hoc implementation of these tools [39] and the usage gap between experienced providers or those working in urban or well-resourced areas [25,26], targeted educational initiatives have led to marked improvements in implementation [29,33]. Moreover, introducing one tool, e.g., HINE, may contribute to increased familiarity with other recommended assessments such as GMA and AIMS, suggesting positive ripple effects [35]. Adoption of other motor assessments tools with strong or conditional recommendation [8]—AIMS, TIMP, DAYC, MAI, or NSMDA—appears to be influenced by training availability, the child’s age, national or institutional preferences, and context-adapted assessments—e.g., nationally validated tools like AIMS for the Spanish population [40].

However, while education and training are critical to strengthen capability, broader efforts are needed to create and sustain opportunities for routine implementation of these tools. Within the CFIR framework, such opportunity-related challenges predominantly map to the “Inner Setting” construct—particularly, available resources and work infrastructure, mirroring findings from related CP implementation studies [41]. A recent example comes from the Ei3 initiative in Los Angeles (US) [32], which strategically combined awareness-raising, alignment with policy requirements, and the co-creation of caregiver-facing materials with iterative quality improvement processes. To address capacity building considering system-level barriers, HINE and basic and advanced GMA trainings were developed, providing scholarships for local care providers and collecting pre/post-training data to assess the impact on provider confidence and knowledge, organizing after an implementation conference.

- From Vision to Routine: Shaping Systems that Make Early Detection the Norm

Integrating evidence-based practices cannot rely solely on individual tools use or technical knowledge. Implementation efforts remain slow and complex [16], and studies in health sciences have documented an average lag of 17 years in translating research into practice [42,43]. Findings across settings highlight that sustaining practice change requires a cultural and organizational shift at the system level. Key enablers include engaging local opinion leaders and establishing communities of practice but also broader structural support such as peer review, ongoing support and supervision, and mechanisms for audit and feedback [44,45]. Crucially, making early detection “the norm” requires cultural change: fostering confidence among providers to communicate risk and guide families, supported by clear protocols [46] and system backing.

Evidence from the CP Early Detection Implementation Network demonstrates the feasibility of maintaining early diagnosis targets over time by adapting to evolving conditions, even under significant stressors such as the COVID-19 pandemic [18]. The standardized telehealth protocol for early detection assessment [47] and the introduction of a new shared metric—the “high-risk” of CP designation to support communication and shared decision-making [48]—illustrate how sustainability depends on feedback and adjustment, mechanisms for distributed innovation, and a shared commitment across the network.

Integrating recommended tools into national child health policies, electronic medical records, and standardized care pathways across all levels of care is a cornerstone. However, challenges remain, e.g., increased time demands because of documentation or lack of interoperable electronic records can reduce access to previously performed assessments (e.g., GMA in NICU), resulting in missing data and unnecessary duplication [29,33].

Together, these actions reflect a shift from isolated tool training to systemic implementation strategies, reinforcing the need for integrated efforts that target multiple domains of the COM-B model and CFIR. International experiences reinforce that full adherence to key recommendations and phases [19]—combined with flexibility to adapt to local contexts—is essential to drive meaningful impact. Conversely, fragmented or partial implementation may fall short of improving core outcomes, such as reducing the average age of CP diagnosis or the risk of continued use of outdated or non-recommended assessments—such as Vojta-based methods [25]—further distancing clinical practice from international standards.

- Aligning Pathways: A Shared Aim for Timely Diagnosis

Nearly half of children diagnosed with CP are born at term and receive routine care at birth [49], making them especially vulnerable to missed or delayed identification when systematic screening is absent, underscoring the urgent need to expand early detection efforts to all at-risk infants [50,51]. In this context, universal CP screening offers a promising solution—including innovative methods like Artificial Intelligence-enhanced GMA [52] and screening tools such as the Brief-HINE [53].

A major barrier that could hinder the integration of these innovations, however, is the lack of clear and standardized referral protocols. Current pathways often are highly dependent on individual clinical discretion or site-specific policies [13,25,28]. This variability contributes to inconsistencies in detection and unequal access to services. Although clinical signs such as stiffness in legs, excessive head lag, or persisting fisting are commonly assessed and recognized by providers [28], they may be identified too late due to their subjective evaluation, evolving presentation, or not triggering referrals because of uncertainty or lack of pathway clarity [50].

To address this, site-specific referral protocols aligned with international guidelines must be standardized and include clear criteria to enhance clinical decision-making across diverse settings and backgrounds, improve communication among health professionals, and promote equity across diverse health systems. Countries such as Spain would particularly benefit from aligning national protocols—from the Spanish Pediatrics Association and the Spanish Society of Neurology [54], Spanish Society of Neonatology [55], and Society of Pediatric Rehabilitation [56]—with international standards to reduce fragmentation and promote cohesive pathways. Shared pathways reflect a shared aim: enabling all infants and families—regardless of where they are born or who assesses them—to access timely, accurate diagnosis and early intervention.

- “If we don’t make an effort to make this whole thing equitable then it won’t be”: Implementation under the Equity Lens

Equity has been identified as a guiding principle in the implementation of early CP detection strategies, particularly considering the challenges faced in low- and middle-income areas [34,57]. These include a higher prevalence of encephalopathies or neonatal infections, together with pronounced structural healthcare system constraints and limited human or technical resources [58].

Context-adapted strategies in Bangladesh led to a 32% diagnostic rate at 12 months (32%) [59]—compared to a national average age of diagnosis of five years [58]—demonstrating the transformative potential of international guidelines implementation. Yet, despite other recent successful implementations in China and Sri Lanka [60,61], critical disparities remain—e.g., in the availability of MRI. In Brazil, neuroimaging referrals accounted for only 1% of early detection cases [26], and in Bangladesh and Sri Lanka, none of the infants had MRI access [59,61].

Variability in access, timing, image quality, and the availability of expert neuroradiological interpretation continues to challenge the global applicability of neuroimaging—raising concerns about imaging-based definitions and early diagnosis pathways [14]. In contexts where MRI is unavailable or unsafe, HINE has been recommended [8] and implemented [59,61]. While cranial ultrasound is less sensitive than MRI—particularly for detecting white matter changes at term [62]—it remains a justified first-line option due to its lower cost, bedside availability, and broader accessibility in low-resource settings.

To promote equity, implementation science must actively embrace and adapt to context. Joint international efforts—such as the BORNTOGETTHERE program, which integrated knowledge translation and guidelines implementation across Italy, Denmark, Netherlands, Georgia, Sri Lanka, and Australia [63]—and a growing body of literature highlight strategies tailored to resource-limited settings. These include, e.g., community-based early intervention and habilitation models [64] and digital tools for remote consultation and scoring [65]. Locally led service innovations and provider capacity-building efforts have shown promise in implementing and expanding early CP detection in low- and middle-income countries [59,60,61].

This review highlights the globally relevant and persistent “know–do gap” in the implementation of early detection guidelines for cerebral palsy. Although tools such as GMA, HINE, and MRI are supported by strong evidence and widely endorsed in international recommendations, their routine use remains limited and uneven across countries. Notably, heterogeneous survey methodologies and predominantly high-resource settings may limit the generalizability of findings to low-resource settings. Additionally, variations in reporting practices and study timeframes constrain direct comparisons.

Our findings suggest that bridging this gap requires more than awareness or training—it demands structural investment, leadership engagement, and system-wide cultural change. Embedding early detection into routine care involves aligning policies, care pathways, and professional practices with context-sensitive implementation strategies. Encouraging more countries to collect baseline data, monitor tool usage rates, and systematically assess local enablers and barriers will be essential for designing tailored plans that ensure these tools are not only available but meaningfully used. Moving from isolated efforts to sustainable, equitable systems will require coordinated action—but the global experiences synthesized in this review show that it is both possible and urgently needed.

## Figures and Tables

**Table 1:** Reported use of recommended and alternative assessment tools based on individual provider reports.

Metric Type	First Author, Year	Providers (*n*)	Use of Recommended Tools (*n*, %)				Use of Alternative Tools (*n*, %)
			GMA	HINE	MRI (Used/Referred)	Other Recommended Assessments	
Individual provider reports	Gmmash, 2019 [23]	269	4 (1% 🔴)	2 (0.7% 🔴)	1 (0.4% 🔴)	DAYC: 33 (12% 🔴) TIMP: 9 (3% 🔴)	Abnormal Involuntary Movement Scale: 23 (8%) AEPS: 6 (2%) Bayley-III: 12 (4%) Battelle: 23 (8%) GMFM 88 and 66: 45 (17%) HELP: 13 (5%) IMP: 12 (4%) PDMS: 68 (25%) Do not use standardized tools: 15 (6%)
	Williams, 2021 [13]	54 (providing diagnosis)	*Children < 1 year*				
			^++^ 12 (25% 🟡) ^+^ 13 (27%) ^−^ 23 (48%)	^++^ 7 (15% 🔴) ^+^ 18 (38%) ^−^ 23 (48%)	^++^ 22 (46% 🟡) ^+^ 23 (48%) ^−^ 3 (6%)	AIMS: ^++^ 6 (13% 🔴), ^+^ 11 (23%), ^−^ 31 (65%) DAYC: ^++^ 17 (35% 🟡), ^+^ 3 (6%), ^−^ 28 (58%) MAI: ^++^ 8, (17% 🔴), ^+^ 7, (15%), ^−^ 33, (69%) NMSDA: ^++^ 3 (6% 🔴), ^+^ 6 (13%), ^−^ 39 (81%) TIMP: ^++^ 3 (6% 🔴), ^+^ 6 (13%), ^−^ 39 (81%)	Bayley: ^++^ 3 (6%), ^+^ 15 (38%), ^−^ 27 (56%) Clinical signs and symptoms: ^++^ 47 (98%), ^−^ 1 (2%) CUS: ^++^ 9 (19%), ^+^ 26 (54%), ^−^ 13 (27%) Dubowitz: ^++^ 2 (4%), ^+^ 16 (33%), ^−^ 30, (63%) Touwen: ^++^ 1 (2%), ^+^ 3 (6%), ^−^ 44 (92%)
			*Children between 1 and 2 years*				
			NA	^++^ 7 (15% 🔴) ^+^ 13 (28%) ^−^ 26 (57%)	^++^ 27 (59% 🟢) ^+^ 15 (33%) ^−^ 4 (9%)	AIMS: ^++^ 7 (15% 🔴), ^+^ 19 (41%), ^−^ 20 (43%) DAYC: ^++^ 16 (35% 🟡), ^+^ 5 (11%), ^−^ 25 (54%) MAI: ^++^ 6 (13% 🔴), ^+^ 7 (15%), ^−^ 33 (72%) NMSDA: ^++^ 3 (7% 🔴), ^+^ 7 (15%), ^−^ 36 (78%) TIMP: NA	Bayley: ^++^ 7 (15%), ^+^ 19 (41%), ^−^ 20 (43%) Clinical signs and symptoms: ^++^ 42 (91%), ^+^ 3 (7%), ^−^ 1 (2%) CUS: ^++^ 4 (9%), ^+^ 11 (24%), ^−^ 31 (67%) Touwen: ^+^ 3 (7%), ^−^ 43 (93%) Dubowitz: NA
			*Children > 2 years*				
			NA	NA	^++^ 21 (55% 🟢) ^+^ 15 (39%) ^−^ 2 (5%)	AIMS: ^+^ 3 (8% 🔴), ^−^ 35 (92%) DAYC: ^++^ 14 (37% 🟡), ^+^ 5 (13%), ^−^ 19 (50%) MAI, NSMDA, TIMP: NA	Bayley: ^++^ 3 (8%), ^+^ 18 (47%), ^−^ 17 (45%) Clinical signs and symptoms: ^++^ 34, (89%), ^+^ 2 (5%), ^−^ 2 (5%) CUS: ^++^ 1 (3%), ^+^ 2 (5%), ^−^ 35 (92%) Touwen: ^++^ 1 (3%), ^+^ 1 (3%), ^−^ 36 (95%) Dubowitz: NA
		104 (not providing diagnosis)	^++^ 15 (14% 🔴) ^+^ 15, (14%) ^−^ 77 (74%)	^++^ 14 (13% 🔴) ^+^ 37, (36%) ^−^ 53 (51%)	^++^ 21 (20% 🔴) ^+^ 24 (23%) ^−^ 59 (57%)	AIMS: ^++^ 20 (19% 🔴), ^+^ 29 (28%), ^−^ 55, (53%) DAYC: ^++^ 17 (16% 🔴), ^+^ 9 (9%), ^−^ 78, (75%) MAI: ^++^ 13 (13% 🔴), ^+^ 15 (14%), ^−^ 76 (73%) NSDA: ^++^ 15 (14% 🔴), ^+^ 15 (14%), ^−^ 84 (81%) TIMP: ^++^ 4 (4% 🔴), ^+^ 4 (4%), ^−^ 100 (96%)	Bayley: ^++^ 13 (13%), ^+^ 30 (29%), ^−^ 61 (59%) Clinical signs and symptoms: ^++^ 90 (87%), ^+^ 12 (12%), ^−^ 2 (2%) CUS: ^++^ 6 (6%), ^+^ 6 (6%), ^−^ 92 (88%) Dubowitz: ^++^ 3 (3%), ^+^ 11 (11%), ^−^ 90, (87%) Touwen: ^−^ 104 (100%)
	Merino-Andrés, 2022 [25]	109	^++^ (25.7% 🟡) ^+^ (11.9%) ^−^ (62.4%)	^++^ (28.4% 🟡) ^+^ (11.9%) ^−^ (59.6%)		AIMS: ^++^ (41.3% 🟡), ^+^ (29.3%), ^−^ (29.3%) DAYC: ^++^ (0.9% 🔴), ^+^ (3.7%), ^−^ (95.4%) MAI: ^++^ (0.9% 🔴), ^+^ (11.9%), ^−^ (87.2%) NSMDA: ^++^ (0.9% 🔴), ^+^ (3.7%), ^−^ (95.4%) TIMP: ^++^ (2.8% 🔴), ^+^ (11%), ^−^ (86.2%)	ASQ: ^++^ (16.5%), ^+^ (13.8%), ^−^ (69.7%) Bayley: ^++^ (12.8%), ^+^ (19.3%), ^−^ (67.9%) Clinical history: ^++^ (88.1%), ^+^ (8.3%), ^−^ (3.7%) Dubowitz: ^++^ (0%), ^+^ (2.8%), ^−^ (97.2%) Touwen: ^++^ (0.9%), ^+^ (2.8%), ^−^ (96.3%) Vojta: ^++^ (32.1%), ^+^ (27.5%), ^−^ (40.4%)
	Hornby, 2024 [28]	72	^++^ (6% 🔴) ^+^ (4%) ^−^ (87%) NA (2%)	^++^ (9% 🔴) ^+^ (20%) ^−^ (70%) NA (2%)	^−^ (*n* = 40, 70.2%)	AIMS: ^++^ (9% 🔴), ^+^ (19%), ^−^ (71%), NA (2%) DAYC: ^++^ (59% 🟢), ^+^ (14%). ^−^ (27%) MAI: ^++^ (12% 🔴), ^+^ (14%), ^−^ (72%), NA (2%) NSMDA: ^++^ (6% 🔴), ^+^ (4%), ^−^ (89%), NA (2%) TIMP: ^++^ (2% 🔴), ^+^ (12%), ^−^ (83%), NA (3%)	Bayley: ^++^ (4%), ^+^ (14%), ^−^ (82%) Dubowitz: ^++^ (2%), ^+^ (4%), ^−^ (91%), NA (4%) PDMS: ^++^ (13%), ^+^ (46%), ^−^ (39%), NA (2%)
	Souza, 2024 [26]	205	55 (26.8% 🟡)	76 (37.1% 🟡)		AIMS: 128 (62.4% 🟢) TIMP: 51 (24.9% 🟡) DAYC: 6 (2.9% 🔴) NSMDA: 19 (9.3% 🔴) None of the options: 51 (24.9%)	

Color codes represent the individual provider-reported level of tool use: red 🔴 (<25%), yellow 🟡 (25–50%, inclusive), and green 🟢 (>50%). Frequency of use: ^++^ Almost always/Several times a week, ^+^ Sometimes/Several times a month/year, ^−^ Never. NA = assessment not applicable to the age range described or the age group participants work with; AIMS = Alberta Infant Motor Scale; ASQ = Ages and Stages Questionnaire; AEPS = Assessment, Evaluation, and Programming System; CUS = Cranial Ultrasound; DAYC = Developmental Assessment of Young Children; Dubowitz = Dubowitz Neurological Examination; GMA = General Movements Assessment; GMFM = Gross Motor Function Measure; HELP = Hawaii Early Learning Profile; HINE = Hammersmith Infant Neurological Examination; IMP = Infant Motor Profile; MAI = Motor Assessment of Infants; MRI = Magnetic Resonance Imaging; NSMDA = Neurological, Sensory, Motor Developmental Assessment; PDMS = Peabody Developmental Motor Scales; TIMP = Test of Infant Motor Performance; Touwen = Touwen Neurological Examination.

**Table 2:** Reported use of recommended and alternative assessment tools based on institutional (neonatal unit) reports.

Metric Type	First author, Year	Services (*n*)	Use of Recommended Tools (*n*, %)				Use of Alternative Tools (*n*, %)
			GMA	HINE	MRI (Used/Referred)	Other Recommended Assessments	
Neonatal units report	Leyener, 2022 [24]	63	^++^ 7 (11% 🔴) ^+^ 11 (17%) ^+/–^ 9 (14%) ^–^ 36 (57%)	NA	14 (22% 🔴)		BNBAS: 3 (5%) CUS: 26 (41%) HNNE: 4 (6%) Miscellaneous: 6 (10%) Neurological examination according to Michaelis: 10 (16%)
	Marcroft, 2025 (preprint) [27]	145	32 (22% 🔴)	26 (17.9% 🔴)		AIMS: 24 (16.6% 🔴)	Bayley Screening Test: 15 (10.3%) Bayley-II: 5 (3.4%) Bayley-III: 80 (55.2%) Denver II: 3 (2.1%) Griffiths-III: 6 (4.1%) Informal assessment only: 23 (15.9%) NBO: 12 (8.3%) PARCA-R: 52 (35.9%) Schedule of Growing Skills: 38 (26.2%) SDQ: 12 (8.3%) Badger 2-year Questionnaire: 64 (44.1%) Other (including Wechsler and ASQ): 11 (7.6%)

Color codes represent the reported level of tool use at the neonatal unit: red 🔴 (<25%). Frequency of use: ^++^ Almost always/Several times a week, ^+^ Several times a month, ^+/–^ Several times a year, ^−^ Never. NA = assessment not applicable to the age range described or the age group participants work with; AIMS = Alberta Infant Motor Scale; ASQ = Ages and Stages Questionnaire; BNBAS = Brazelton Neonatal Behavioral Assessment Scale; CUS = Cranial Ultrasound; Denver II = Denver Developmental Screening Test II; GMA = General Movements Assessment; Griffiths-III = Griffiths Mental Development Scales, Third Edition; HINE = Hammersmith Infant Neurological Examination; HNNE = Hammersmith Neonatal Neurological Examination; MRI = Magnetic Resonance Imaging; NBO = Newborn Behavioral Observations; PARCA-R = Parent Report of Children’s Abilities – Revised; SDQ = Strengths and Difficulties Questionnaire.

**Table 3:** Reported use of recommended assessment tools based on patient-level data (clinical records).

Metric Type	First Author, Year	Infants (*n*)	Use of Recommended Tools (*n*, %)			
			GMA	HINE	MRI (Used/Referred)	Other Recommended Assessments
Patient-level data	Maitre, 2016 [29]	50		*Before training* (37% 🟡)		
				*After training* (90% 🟢)		
	Sutter, 2024 [31]	44	*Before guidelines publication* (~5% 🔴)	*Before guidelines publication* (0% 🔴)	*Before guidelines publication* (90% 🟢)	*Before guidelines publication* AIMS (~40% 🟡) TIMP (~10% 🔴) DAYC (0% 🔴)
		47	*After guidelines publication* (~55% 🟢)	*After guidelines publication* (~17% 🔴)	*After guidelines publication* (~92% 🟢)	*After guidelines publication* AIMS (~30% 🟡) TIMP (~50% 🟢) DAYC (~5% 🔴)
	Davidson, 2022 [33]	6	*Pre-implementation:* Writhing/fidgety: 1 (16.7% 🔴) No GMA: 5 (83.3%)	*Pre-implementation (infants referred ≤ 5 months):* ≤5 months: 0 (0% 🔴) >5 months: 0 (0% 🔴) No HINE: 6 (100%)	*Pre-implementation (infants referred ≤ 5 months):* ≤5 months: 2 (33.3% 🟡) >5 months: 1 (16.7% 🔴) No MRI: 3 (50%)	
		209	*Implementation phases:* Writhing/fidgety: 127 (60.8% 🟢) No GMA: 82 (39.2%)	*Implementation phases (infants referred < 5 months):* ≤5 months: 57 (27.3% 🟡) >5 months: 44 (21.1% 🔴) No HINE: 108 (51.7%)	*Implementation phases (infants referred ≤ 5 months):* ≤5 months: 161 (77% 🟢) >5 months: 14 (16.7% 🔴) No MRI: 34 (16.3%)	
		43	NA	*Pre-implementation (infants referred > 5 months):* 0 (0% 🔴) Missing: 27 (62.7%) Not eligible: 16 (37.2%)	*Pre-implementation (infants referred > 5 months):* ≤5 months: 0 (0% 🔴) >5 months: 2 (4.7% 🔴) No MRI: 41 (95.3%)	
		236	NA	*Implementation phases (infants referred > 5 months):* 12 (5.1% 🔴) Missing: 167 (70.8%) Not eligible: 57 (24.2%)	*Implementation phases (infants referred > 5 months):* ≤5 months: 24 (10.2% 🔴) >5 months: 124 (52.5% 🟢) No MRI: 88 (37.3%)	

Color codes represent the reported level of tool use as documented in patient-level data (clinical records): red 🔴 (<25%), yellow 🟡 (25–50%, inclusive), and green 🟢 (>50%). For Sutter, 2024 [31] exact data were not provided in the text; estimated percentages were visually estimated from Figure 1. NA = assessment not applicable to the age range described or the age group participants work with; AIMS = Alberta Infant Motor Scale; DAYC = Developmental Assessment of Young Children; GMA = General Movements Assessment; HINE = Hammersmith Infant Neurological Examination; MRI = Magnetic Resonance Imaging; TIMP = Test of Infant Motor Performance.

**Table 4:** Providers-identified enablers and barriers to implementing recommended assessment tools for early CP detection.

Factors	Enablers (*n*)	Barriers (*n*)
System factors	Time and funding (*n* = 13) [13], (*n* = 7) [25]; System and personnel resources (53 quotes) [34]	Time, workload, and staffing (*n* = 25) [13], (*n* = 46) [25], (*n* not specified, ≤12) [29]; System and personnel resources (149 quotes) [34]; Funding (*n* = 19) [13], (*n* = 28) [25]
	Funding; tool availability and use; time, workload, staffing; organizational structure/processes (*n* = 13) [28]	Funding; tool availability and use; time, workload, staffing; organizational structure/processes (*n* = 44) [28]
		Referral and health pathways (*n* = 12) [13], (*n* = 16) [25]
	Quality improvement, peer review, and audit (*n* = 16) [13]	
	👤👤👤👤👤👤👤👤👤👤 (*n* = 49)	👤👤👤👤👤👤👤👤👤👤 👤👤👤👤👤👤👤👤👤👤 👤👤👤👤👤👤👤👤👤👤 👤👤👤👤👤👤👤👤 (*n* = 190)
Social factors	Management, staff, and administration (*n* = 19) [13], (*n* = 9) [25]	Management, staff, and administration (*n* = 14) [13], (*n* = 12) [25]
	Multidisciplinary teamwork (*n* = 8) [13], (*n* = 28) [25]	Multidisciplinary teamwork (*n* = 8) [13], (*n* = 18) [25]
	Administration/leadership and peer support/multidisciplinary working/clinical champions (*n* = 25) [28] Role of leadership (12 quotes) [34]	Administration/leadership, peer support/multidisciplinary working/clinical champions (*n* = 21) [28]; Role of leadership (10 quotes) [34]
	👤👤👤👤👤👤👤👤👤👤 👤👤👤👤👤👤👤👤 (*n* = 89)	👤👤👤👤👤👤👤👤👤👤 👤👤👤👤👤 (*n* = 73)
Health professional knowledge and perceptions	Education/professional development and knowledge sharing (*n* = 16) [13], (*n* = 22) [25]	Knowledge/confidence in using tools (*n* = 10) [13], (*n* = 21) [25]; Inconsistent knowledge base about time and specifics of the neurological exam because of provider-type diversity (*n* not specified, ≤12) [29]
	Guidelines and clinical pathways (*n* = 6) [13], (*n* = 9) [25]; Consensus about research evidence (11 quotes) [34]	Guidelines and clinical pathways (*n* = 6) [13]; Consensus about research evidence (15 quotes) [34]
	Health professional communication (*n* = 4) [25]	Health professional communication (*n* = 1) [25]
	Patient-tailored care (*n* = 1) [25]	
	Clinical experience (23 quotes) [34]	Clinical experience (52 quotes) [34]
	Family experience as evidence (35 quotes) [34]	Family experience as evidence (61 quotes) [34]
	Evaluation practices (1 quote) [34]	Evaluation practices (14 quotes) [34]
	Access to education; knowledge sharing/confidence/practice opportunities; guidelines and pathways (*n* = 22) [28]	Access to education; knowledge sharing/confidence/practice opportunities; guidelines and pathways (*n* = 19) [28]
	👤👤👤👤👤👤👤👤👤👤 👤👤👤👤👤👤 (*n* = 80)	👤👤👤👤👤👤👤👤👤👤 👤 (*n* = 57)
Clinical considerations and internal drive	Self-driven/self-initiated (*n* = 13) [25]	
		Case complexity and inconsistency in practice (*n* = 5) [13], (*n* = 1) [25]; Concerns about a complex neurological exam decreasing the clinical flow without tangible benefits to patients (*n* not specified, ≤12) [29]
		Physical possibilities (*n* = 7) [25]
	Clinical considerations and internal drive (*n* = 6) [28]	Clinical considerations and internal drive (*n* = 4) [28]
	👤👤👤👤 (*n* = 19)	👤👤👤 (*n* = 17)

Each 👤 symbol represents 5 respondents. For the article by Mulqueeney et al. 2024 [34], enabler/barrier *n* is reported as the number of supporting quotes (not added to the total count of participants to avoid duplication).

## Data Availability

Not applicable.

## Supplementary Materials

The following supporting information can be downloaded at: https://www.mdpi.com/article/10.3390/children12070941/s1, Table S1: PRISMA-ScR checklist item; Table S2: Search strategy; Table S3: Excluded articles and rationale.

## Abbreviations

The following abbreviations are used in this manuscript:

AIMS	Alberta Infant Motor Scale
ASQ	Ages and Stages Questionnaires
CFIR	Consolidated Framework for Implementation Research
CP	Cerebral Palsy
DAYC	Developmental Assessment of Young Children
Ei3	Early Identification and Intervention for Infants Network
GMA	General Movement Assessment
HINE	Hammersmith Infant Neurological Examination
JBI	Joanna Briggs Institute
MAI	Motor Assessment of Infants
MRI	Magnetic Resonance Imaging
NGO	Non-Governmental Organizations
NICU	Neonatal Intensive Care Unit
NSMDA	Neuro-Sensory Motor Developmental Assessment
PCC	Participant–Concept–Context
PDMS-2	Peabody Developmental Motor Scales
PRISMA-ScR	Preferred Reporting Items for Systematic Reviews and Meta-Analyses Extension for Scoping Reviews
TIMP	Test of Infant Motor Performance
US	United States

## Appendix A

**Table A1:** Characteristics of included articles.

First Author, Year	Country	Method	*n*	Setting (*n*, %)	Providers (*n*, %)	Experience
Maitre, 2016 [29]	US	Survey and electronic medical record audit	12 (survey) and 50 electronic medical records	Nationwide Children’s Hospital (Neonatal follow-up program)	Advance practice nurses, general pediatricians, developmental/behavioral pediatricians, pediatric neurologist, neonatologists, and specialty fellows in neonatology and developmental medicine	Not described
Byrne, 2017 [30]	US	Survey	11–26	Nationwide Children’s Hospital (Neonatal follow-up program)	Physical therapist, occupational therapist, advanced nurse practitioners, general pediatricians, developmental pediatricians, pediatric neurologists, nurses, dietitians, social workers, and speech–language therapist	Not described
Gmmash, 2019 [23]	US	Survey	269	Early intervention	Physical therapists (*n* = 180) and occupational therapists (*n* = 89)	Not described
Williams, 2021 [13]	New Zealand	Survey	159	Hospital/health services (*n* = 116, 77%), private practice (*n* = 7, 5%), hospital/private practice (*n* = 9, 6%), university (*n* = 5, 3%), non-governmental organization (*n* = 4, 3%)	Physical therapists (*n* = 66), occupational therapists (*n* = 23), general pediatricians (*n* = 22), neurodevelopmental therapists (*n* = 11), neonatologists (*n* = 10), orthopedic surgeons (*n* = 7), developmental pediatricians (*n* = 5), trainee doctors (*n* = 4), speech–language therapists (*n* = 4), general practitioners (*n* = 2), pediatric rehabilitation consultant (*n* = 1), nurse specialist (*n* = 1), and early childhood nurse (*n* = 1)	1–5 years (*n* = 22, 15%), 6–14 years (*n* = 52, 35%), 15+ years (*n* = 75, 50%)
Davidson, 2022 [33]	Australia	Pre/post-implementation data	Not reported	Statewide tertiary pediatric early intervention service (Western Australia)	Not described	Not described
Leyener, 2022 [24]	Germany	Survey	Not reported	NICUs	Medical doctors, physical therapists, nurses, and others	Not described
Merino-Andrés, 2022 [25]	Spain	Survey	109	Early intervention (infants < 1 year): clinical centers (62.9%)	Physical therapists (*n* = 109, 100%)	<10 years (58.6%)
Butera, 2024 [32]	US	Implementation (awareness and capacity-building), pre/post-training survey	70 (symposium), 211 (HINE training), 46 (GMA training), Implementation conference	Early Identification and Intervention for Infants Network, Los Angeles (participants of HINE training): children’s services medical therapy program (51.1%), children’s services general program (1.4%), NICU (7.2%), inpatient pediatrics (10.1%), outpatient therapy practice (16.5%), outpatient medical practice (2.9%), early intervention (6.5%)	Participants of HINE training: physical therapists (*n* = 114, 64.4%), occupational therapists (*n* = 44, 24.9%), developmental pediatrician (*n* = 4, 2.3%), pediatrics (*n* = 4, 2.3%), nurse practitioner (*n* = 4, 2.3%), speech–language therapists (*n* = 2, 1.1%), and neonatology (*n* = 2, 1.1%)	Not described
Hidalgo-Robles, 2024 [35]	Spain	Pre/post-training survey	11	Early intervention	Physical therapists (*n* = 3, 27.3%), speech–language therapists (*n* = 3, 27.3%), psychologists (*n* = 3, 27.3%), and occupational therapists (*n* = 2, 18.2%)	<5 years (54%), 5–15 years (36%), 16–20 years (9%)
Hornby, 2024 [28]	US	Survey	72	Maryland and Delaware Early Intervention: Health or education settings for children < 3 years, with risk factors for CP	Physical therapists (*n* = 36, 48.6%), occupational therapists (*n* = 18, 24.3%), developmental pediatrician (*n* = 1, 1.4%), general pediatrician (*n* = 3, 4.1%), orthopedic surgeon (*n* = 1, 1.4%), pediatric neurologist (*n* = 3, 4.1%), physiatrist (*n* = 1, 1.4%), medically complex pediatrician (*n* = 2, 2.7%), nurse practitioner/physician assistant (*n* = 1, 1.4%), early childhood nurse (*n* = 2, 2.7%), researcher (*n* = 1, 1.4%), early intervention administrator (*n* = 1, 1.4%), social worker (*n* = 1, 1.4%), special educator (*n* = 2, 2.7%), and speech–language therapist (*n* = 1, 1.4%)	6–14 years (*n* = 21, 29.6%), 15+ years (*n* = 50, 70.4%)
Mulqueeney, 2024 [34]	New Zealand	Survey, focus groups	27 (survey), 20 (focus groups/interviews)	Te Toka Tumai Auckland NICU, Starship Children’s Hospital community, developmental pediatrics services, Auckland	Nurses (*n* = 13, 48%), neonatologists (*n* = 7, 26%), therapists (occupational therapist, physical therapist, speech therapist) (*n* = 4, 15%), developmental pediatricians (*n* = 2, 7%), and neonatal registrar (*n* = 1, <1%)	1–3 years (*n* = 3, 11%), 4–10 (*n* = 3, 11%), +10 years (*n* = 21, 78%)
Souza, 2024 [26]	Brazil	Survey	205	Early intervention (< 3 years): public health services (77, 37.6%), private healthcare providers (71, 34.6%), universities and other educational institutions (28, 13.7%), self-employed (21, 10.2%), NGO (5, 2.4%), other services (3, 1.5%)	Physical therapists (*n* = 168, 82%) and occupational therapists (*n* = 37, 18%)	0–5 years (*n* = 21, 10.2%), 5–10 years (*n* = 46, 22.4%), 10–15 years (*n* = 47, 22.9%), 15–20 years (*n* = 34, 16.6%), >20 years (*n* = 53, 25.9%)
Sutter, 2024 [31]	US	Pre/postguidelines-publication retrospective medical record review	Not reported	University of Wisconsin Waisman Center Newborn Follow-Up Clinic, Madison (Newborn Follow-Up clinic < 3 years)	Developmental pediatricians, pediatric neurologists, physical medicine rehabilitation consultants, physical therapists, occupational therapists, and speech–language therapists	Not described
Marcroft, 2025 [27]	UK (England, Scotland, and Wales)	Survey (preprint)	154	Neonatal units (NICUs, Local Neonatal Units, Special Care Baby Units)	Medical doctors (*n* = 124, 80.5%), physical therapists (*n* = 22, 14.3%), occupational therapists (*n* = 4, 2.6%), and nurses/other (*n* = 4, 2.6%)	Not described

NGO = non-governmental organizations; NICU = Neonatal Intensive Care Unit; US = United States.
