# Supplementary material for: The Pathway Is Clear but the Road Remains Unpaved: A Scoping Review of Implementation of Tools for Early Detection of Cerebral Palsy
Source: Children (Basel). 2025 Jul 17;12(7):941. doi: 10.3390/children12070941 (PMC12293170; doi:10.3390/children12070941)
Supplement: Supplementary file 1 [file children-12-00941-s001.zip › children-3708190-supplementary.pdf]

Table S1. Preferred Reporting Items for Systematic reviews and Meta-Analyses extension for Scoping Reviews (PRISMA-ScR) Checklist

SECTION	ITEM	PRISMA-ScR CHECKLIST ITEM	REPORTED ON PAGE #
**TITLE**			
Title	1	Identify the report as a scoping review.	1
**ABSTRACT**			
Structured summary	2	Provide a structured summary that includes (as applicable): background, objectives, eligibility criteria, sources of evidence, charting methods, results, and conclusions that relate to the review questions and objectives.	1
**INTRODUCTION**			
Rationale	3	Describe the rationale for the review in the context of what is already known. Explain why the review questions/objectives lend themselves to a scoping review approach.	1-2
Objectives	4	Provide an explicit statement of the questions and objectives being addressed with reference to their key elements (e.g., population or participants, concepts, and context) or other relevant key elements used to conceptualize the review questions and/or objectives.	2
**METHODS**			
Protocol and registration	5	Indicate whether a review protocol exists; state if and where it can be accessed (e.g., a Web address); and if available, provide registration information, including the registration number.	2
Eligibility criteria	6	Specify characteristics of the sources of evidence used as eligibility criteria (e.g., years considered, language, and publication status), and provide a rationale.	3
Information sources*	7	Describe all information sources in the search (e.g., databases with dates of coverage and contact with authors to identify additional sources), as well as the date the most recent search was executed.	2-3
Search	8	Present the full electronic search strategy for at least 1 database, including any limits used, such that it could be repeated.	Table S1
Selection of sources of evidence†	9	State the process for selecting sources of evidence (i.e., screening and eligibility) included in the scoping review.	3
Data charting process‡	10	Describe the methods of charting data from the included sources of evidence (e.g., calibrated forms or forms that have been tested by the team before their use, and whether data charting was done independently or in duplicate) and any processes for obtaining and confirming data from investigators.	3-4
Data items	11	List and define all variables for which data were sought and any assumptions and simplifications made.	3
Critical appraisal of individual sources of evidence§	12	If done, provide a rationale for conducting a critical appraisal of included sources of evidence; describe the methods used and how this information was used in any data synthesis (if appropriate).	-
Synthesis of results	13	Describe the methods of handling and summarizing the data that were charted.	3-4

SECTION	ITEM	PRISMA-ScR CHECKLIST ITEM	REPORTED ON PAGE #
**RESULTS**			
Selection of sources of evidence	14	Give numbers of sources of evidence screened, assessed for eligibility, and included in the review, with reasons for exclusions at each stage, ideally using a flow diagram.	4
Characteristics of sources of evidence	15	For each source of evidence, present characteristics for which data were charted and provide the citations.	4-12
Critical appraisal within sources of evidence	16	If done, present data on critical appraisal of included sources of evidence (see item 12).	-
Results of individual sources of evidence	17	For each included source of evidence, present the relevant data that were charted that relate to the review questions and objectives.	4-12
Synthesis of results	18	Summarize and/or present the charting results as they relate to the review questions and objectives.	Table 1-4, Appendix A
**DISCUSSION**			
Summary of evidence	19	Summarize the main results (including an overview of concepts, themes, and types of evidence available), link to the review questions and objectives, and consider the relevance to key groups.	13
Limitations	20	Discuss the limitations of the scoping review process.	15
Conclusions	21	Provide a general interpretation of the results with respect to the review questions and objectives, as well as potential implications and/or next steps.	15
**FUNDING**			
Funding	22	Describe sources of funding for the included sources of evidence, as well as sources of funding for the scoping review. Describe the role of the funders of the scoping review.	16

JBI = Joanna Briggs Institute; PRISMA-ScR = Preferred Reporting Items for Systematic reviews and Meta-Analyses extension for Scoping Reviews.
* Where *sources of evidence* (see second footnote) are compiled from, such as bibliographic databases, social media platforms, and Web sites.
† A more inclusive/heterogeneous term used to account for the different types of evidence or data sources (e.g., quantitative and/or qualitative research, expert opinion, and policy documents) that may be eligible in a scoping review as opposed to only studies. This is not to be confused with *information sources* (see first footnote).
‡ The frameworks by Arksey and O'Malley (6) and Levac and colleagues (7) and the JBI guidance (4, 5) refer to the process of data extraction in a scoping review as data charting.
§ The process of systematically examining research evidence to assess its validity, results, and relevance before using it to inform a decision. This term is used for items 12 and 19 instead of "risk of bias" (which is more applicable to systematic reviews of interventions) to include and acknowledge the various sources of evidence that may be used in a scoping review (e.g., quantitative and/or qualitative research, expert opinion, and policy document).

From: Tricco AC, Lillie E, Zarin W, O'Brien KK, Colquhoun H, Levac D, et al. PRISMA Extension for Scoping Reviews (PRISMAScR): Checklist and Explanation. Ann Intern Med. 2018;169:467–473. doi: 10.7326/M18-0850.

Table S2. Search strategy

Database	Search string
**PubMed**	("cerebral palsy"[MeSH Terms] OR "cerebral palsy"[Title/Abstract] OR "CP"[Title/Abstract]) AND ("diagnosis"[Title/Abstract] OR "early diagnosis"[MeSH Terms] OR "early detection"[Title/Abstract] OR "early diagnosis"[Title/Abstract] OR "early identification"[Title/Abstract]) AND ("General Movements Assessment"[Title/Abstract] OR "Prechtl"[Title/Abstract] OR "GMA"[Title/Abstract] OR "Hammersmith Infant Neurological Examination"[Title/Abstract] OR "HINE"[Title/Abstract] OR "neuroimaging"[Title/Abstract] OR "magnetic resonance imaging"[Title/Abstract] OR "MRI"[Title/Abstract] OR "cranial ultrasound"[Title/Abstract]) AND ("implementation"[Title/Abstract] OR "uptake"[Title/Abstract] OR "adoption"[Title/Abstract] OR "integration"[Title/Abstract] OR "barriers"[Title/Abstract] OR "facilitators"[Title/Abstract] OR "feasibility"[Title/Abstract] OR "acceptability"[Title/Abstract] OR "clinical practice"[Title/Abstract] OR "healthcare system"[Title/Abstract] OR "survey"[Title/Abstract] OR "questionnaire"[Title/Abstract] OR "interview"[Title/Abstract] OR "perception"[Title/Abstract] OR "attitudes"[Title/Abstract])
**ProQuest**	TI,AB("cerebral palsy" OR "CP") AND TI,AB("diagnosis" OR "early diagnosis" OR "early detection" OR "early identification") AND TI,AB("General Movements Assessment" OR "Prechtl" OR "GMA" OR "Hammersmith Infant Neurological Examination" OR "HINE" OR "neuroimaging" OR "magnetic resonance imaging" OR "MRI" OR "cranial ultrasound") AND TI,AB("implementation" OR "uptake" OR "adoption" OR "integration" OR "barriers" OR "facilitators" OR "feasibility" OR "acceptability" OR "clinical practice" OR "healthcare system" OR "survey" OR "questionnaire" OR "interview" OR "perception" OR "attitudes")
**Web of Science**	TS=("cerebral palsy" OR "CP") AND TS=("diagnosis" OR "early diagnosis" OR "early detection" OR "early identification") AND TS=("General Movements Assessment" OR "Prechtl" OR "GMA" OR "Hammersmith Infant Neurological Examination" OR "HINE" OR "neuroimaging" OR "magnetic resonance imaging" OR "MRI" OR "cranial ultrasound") AND TS=("implementation" OR "uptake" OR "adoption" OR "integration" OR "barriers" OR "facilitators" OR "feasibility" OR "acceptability" OR "clinical practice" OR "healthcare system" OR "survey" OR "questionnaire" OR "interview" OR "perception" OR "attitudes")
**PEDro**	Cerebral palsy AND diagnosis
**Cochrane Library**	(cerebral palsy):ti,ab,kw AND (diagnosis OR "early diagnosis" OR "early detection" OR "early identification"):ti,ab,kw AND ("General Movements Assessment" OR Prechtl OR GMA OR "Hammersmith Infant Neurological Examination" OR HINE OR neuroimaging OR "magnetic resonance imaging" OR MRI OR "cranial ultrasound"):ti,ab,kw AND (implementation OR uptake OR adoption OR integration OR barriers OR facilitators OR feasibility OR acceptability OR "clinical practice" OR "healthcare system" OR survey OR questionnaire OR interview OR perception OR attitudes):ti,ab,kw
**Scopus**	(TITLE-ABS-KEY ("cerebral palsy") OR TITLE-ABS-KEY ("CP")) AND (TITLE-ABS-KEY ("diagnosis") OR TITLE-ABS-KEY ("early diagnosis") OR TITLE-ABS-KEY ("early detection") OR TITLE-ABS-KEY ("early identification")) AND (TITLE-ABS-KEY ("General Movements Assessment") OR TITLE-ABS-KEY ("Prechtl") OR TITLE-ABS-KEY ("GMA") OR TITLE-ABS-KEY ("Hammersmith Infant Neurological Examination") OR TITLE-ABS-KEY ("HINE") OR TITLE-ABS-KEY ("neuroimaging") OR TITLE-ABS-KEY ("magnetic resonance imaging") OR TITLE-ABS-KEY ("MRI") OR TITLE-ABS-KEY ("cranial

Database	Search string
	ultrasound")) AND (TITLE-ABS-KEY ("implementation") OR TITLE-ABS-KEY ("uptake") OR TITLE-ABS-KEY ("adoption") OR TITLE-ABS-KEY ("integration") OR TITLE-ABS-KEY ("barriers") OR TITLE-ABS-KEY ("facilitators") OR TITLE-ABS-KEY ("feasibility") OR TITLE-ABS-KEY ("acceptability") OR TITLE-ABS-KEY ("clinical practice") OR TITLE-ABS-KEY ("healthcare system") OR TITLE-ABS-KEY ("survey") OR TITLE-ABS-KEY ("questionnaire") OR TITLE-ABS-KEY ("interview") OR TITLE-ABS-KEY ("perception") OR TITLE-ABS-KEY ("attitudes"))

Table S3. Excluded articles and rationale.

Bonouvrié, L. A., Becher, J. G., Vles, J. S., Boeschoten, K., Soudant, D., de Groot, V., van Ouwerkerk, W. J., Strijers, R., Foncke, E., Geytenbeek, J., van de Ven, P. M., Teernstra, O., & Vermeulen, R. J. (2013). Intrathecal baclofen treatment in dystonic cerebral palsy: a randomized clinical trial: the IDYS trial. BMC pediatrics, 13, 175. https://doi.org/10.1186/1471-2431-13-175	Wrong outcome
Gmmash AS, Effgen SK, Goldey K. Challenges Faced by Therapists Providing Services for Infants With or at Risk for Cerebral Palsy. Pediatr Phys Ther. 2020 Apr;32(2):88-96. doi: 10.1097/PEP.0000000000000686. PMID: 32150028.	Wrong outcome
Kleeren, L., Mailleux, L., McLean, B., Elliott, C., Dequeker, G., Van Campenhout, A., de Xivry, J. O., Verheyden, G., Ortibus, E., Klingels, K., & Feys, H. (2024). Does somatosensory discrimination therapy alter sensorimotor upper limb function differently compared to motor therapy in children and adolescents with unilateral cerebral palsy: study protocol for a randomized controlled trial. Trials, 25(1), 147. https://doi.org/10.1186/s13063-024-07967-4	Wrong outcome
Fujimoto S, Togari H, Takashima S, Funato M, Yoshioka H, Ibara S, Tatsuno M. National survey of periventricular leukomalacia in Japan. Acta Paediatr Jpn. 1998 Jun;40(3):239-43. doi: 10.1111/j.1442-200x.1998.tb01919.x. PMID: 9695297.	Wrong outcome
Baggaley J, Seiboth C, Rapley T, Basu A. From eligibility to diagnosis: candidacy and the complex journey of cerebral palsy diagnosis within primary care. BMC Pediatr. 2025 Feb 12;25(1):112. doi: 10.1186/s12887-025-05455-5. PMID: 39939861; PMCID: PMC11823032.	Wrong outcome
Barišić, N., Prpić, I., Lehman, I., Grđan, P., & Rešić, B. (2012). The Croatian Society of pediatric neurology guidelines for diagnosis and treatment of headaches in children. Paediatria Croatica, 56(2), 147-155. https://doi.org/10.13112/pc.749	Wrong outcome

Friedrich, C., Boekhoff, S., Bischoff, M., Beckhaus, J., Sowithayasakul, P., Calaminus, G., Eveslage, M., Valentini, C., Bison, B., Harrabi, S. B., Krause, M., Timmermann, B., & Müller, H. L. (2023). Outcome after proton beam therapy versus photon-based radiation therapy in childhood-onset craniopharyngioma patients-results of KRANIOPHARYNGEOM 2007. Frontiers in oncology, 13, 1180993. https://doi.org/10.3389/fonc.2023.1180993	Wrong population
Austin, T., Shanmugalingam, S., & Clarke, P. (2013). To cool or not to cool? Hypothermia treatment outside trial criteria. Archives of disease in childhood. Fetal and neonatal edition, 98(5), F451–F453. https://doi.org/10.1136/archdischild-2012-302069	Wrong outcome
Hoffmann, G. F., Surtees, R. A., & Wevers, R. A. (1998). Cerebrospinal fluid investigations for neurometabolic disorders. Neuropediatrics, 29(2), 59–71. https://doi.org/10.1055/s-2007-973538	Wrong population
Betancourt, J. P., Eleeh, P., Stark, S., & Jain, N. B. (2019). Impact of Ankle-Foot Orthosis on Gait Efficiency in Ambulatory Children With Cerebral Palsy: A Systematic Review and Meta-analysis. American journal of physical medicine & rehabilitation, 98(9), 759–770. https://doi.org/10.1097/PHM.0000000000001185	Wrong outcome
Adıgüzel, H., Sarıkabadayı, Y. Ü., Apaydın, U., Katırcı Kırmacı, Z. İ., Gücüyener, K., Günel Karadeniz, P., & Elbasan, B. (2022). Turkish Validity and Reliability of the Hammersmith Infant Neurological Examination (HINE) with High-Risk Infant Group: A Preliminary Study. Turkish archives of pediatrics, 57(2), 151–159. https://doi.org/10.5152/TurkArchPediatr.2022.21231	Wrong outcome
Lennon, S., McKenna, S., & Jones, F. (2013). Self-management programmes for people post stroke: a systematic review. Clinical rehabilitation, 27(10), 867–878. https://doi.org/10.1177/0269215513481045	Wrong population

Furtado, M. A. D. S., Leite, H. R., Klettenberg, M. R. P., Rodrigues, V. A., Ferreira, L. S., Marques, M. R., Cavalcante, I. O., Santos, T. S., Souza, T. G., Mendonça, A. S. G. B., Camargos, A. C. R., & Ayupe, K. M. A. (2024). Translation and measurement properties of the Portuguese-Brazil version of the Hammersmith Infant Neurological Examination (HINE-Br). Revista paulista de pediatria : orgao oficial da Sociedade de Pediatria de Sao Paulo, 42, e2023105. https://doi.org/10.1590/1984-0462/2024/42/2023105	Wrong outcome
Byrne, R., Noritz, G., Maitre, N. L., & NCH Early Developmental Group (2017). Implementation of Early Diagnosis and Intervention Guidelines for Cerebral Palsy in a High-Risk Infant Follow-Up Clinic. Pediatric neurology, 76, 66–71. https://doi.org/10.1016/j.pediatrneurol.2017.08.002	Wrong outcome
Barron-Garza, F., Coronado-Garza, M., Gutierrez-Ramirez, S., Ramos-Rincon, J. M., Guzman-de la Garza, F., Lozano-Morantes, A., Flores-Rodriguez, A., Nieto-Sanjuanero, A., Alvarez-Villalobos, N., Flores-Villarreal, M., & Covarrubias-Contreras, L. (2023). Incidence of Cerebral Palsy, Risk Factors, and Neuroimaging in Northeast Mexico. Pediatric neurology, 143, 50–58. https://doi.org/10.1016/j.pediatrneurol.2023.02.005	Wrong outcome
Zoccolillo, L., Morelli, D., Cincotti, F., Muzzioli, L., Gobbetti, T., Paolucci, S., & Iosa, M. (2015). Video-game based therapy performed by children with cerebral palsy: a cross-over randomized controlled trial and a cross-sectional quantitative measure of physical activity. European journal of physical and rehabilitation medicine, 51(6), 669–676.	Wrong outcome
McNamara, L., Scott, K., Boyd, R. N., Farmer, E., Webb, A., Bosanquet, M., Nguyen, K., & Novak, I. (2022). Can web-based implementation interventions improve physician early diagnosis of cerebral palsy? Protocol for a 3-arm parallel superiority randomised controlled trial and cost-consequence analysis comparing adaptive and non-adaptive virtual patient	Wrong outcome

instructional designs with control to evaluate effectiveness on physician behaviour, diagnostic skills and patient outcomes. BMJ open, 12(11), e063558. https://doi.org/10.1136/bmjopen-2022-063558	
Holmström, L., Eliasson, A. C., Almeida, R., Furmark, C., Weiland, A. L., Tedroff, K., & Löwing, K. (2019). Efficacy of the Small Step Program in a Randomized Controlled Trial for Infants under 12 Months Old at Risk of Cerebral Palsy (CP) and Other Neurological Disorders. Journal of clinical medicine, 8(7), 1016. https://doi.org/10.3390/jcm8071016	Wrong outcome
Boyd, R., Sakzewski, L., Ziviani, J., Abbott, D. F., Badawy, R., Gilmore, R., Provan, K., Tournier, J. D., Macdonell, R. A., & Jackson, G. D. (2010). INCITE: A randomised trial comparing constraint induced movement therapy and bimanual training in children with congenital hemiplegia. BMC neurology, 10, 4. https://doi.org/10.1186/1471-2377-10-4	Wrong outcome
Palmer F. B. (2004). Strategies for the early diagnosis of cerebral palsy. The Journal of pediatrics, 145(2 Suppl), S8–S11. https://doi.org/10.1016/j.jpeds.2004.05.016	Wrong outcome
Kwong, A. K. L., Eeles, A. L., Olsen, J. E., Zannino, D., Kariotis, T., & Spittle, A. J. (2022). Instructional guides for filming infant movements at home are effective for the General Movements Assessment. Journal of paediatrics and child health, 58(5), 796–801. https://doi.org/10.1111/jpc.15838	Wrong outcome
Nolan, S. J., Tudur Smith, C., Weston, J., & Marson, A. G. (2016). Lamotrigine versus carbamazepine monotherapy for epilepsy: an individual participant data review. The Cochrane database of systematic reviews, 11(11), CD001031. https://doi.org/10.1002/14651858.CD001031.pub3	Wrong population
Ohlsson, A., & Aher, S. M. (2017). Early erythropoiesis-stimulating agents in preterm or low birth weight infants. The Cochrane database of systematic reviews, 11(11), CD004863.	Wrong population

https://doi.org/10.1002/14651858.CD004863.pub5	
Pollock, A., Farmer, S. E., Brady, M. C., Langhorne, P., Mead, G. E., Mehrholz, J., & van Wijck, F. (2014). Interventions for improving upper limb function after stroke. The Cochrane database of systematic reviews, 2014(11), CD010820. https://doi.org/10.1002/14651858.CD010820.pub2	Wrong population
Zlatanovic, D., Čolović, H., Živković, V., Stanković, A., Kostić, M., Vučić, J., & Tošić, T. (2022). The importance of assessing general motor activity in premature infants for predicting neurological outcomes. Folia neuropathologica, 60(4), 427–435. https://doi.org/10.5114/fn.2022.119593	Wrong outcome
Guttmann, K., Flibotte, J., DeMauro, S. B., & Seitz, H. (2020). A Mixed Methods Analysis of Parental Perspectives on Diagnosis and Prognosis of Neonatal Intensive Care Unit Graduates With Cerebral Palsy. Journal of child neurology, 35(5), 336–343. https://doi.org/10.1177/0883073820901412	Wrong outcome
Fortune, A., Perkins, E., Paize, F., Palanisami, B., & Gladstone, M. (2024). Managing mothers' and fathers' uncertainty during their journey through early neurodevelopmental follow-up for their high-risk infants-A qualitative account. Child: care, health and development, 50(1), e13168. https://doi.org/10.1111/cch.13168	Wrong outcome
Zhang J. (2017). Multivariate Analysis and Machine Learning in Cerebral Palsy Research. Frontiers in neurology, 8, 715. https://doi.org/10.3389/fneur.2017.00715	Wrong outcome
Bruschettini, M., Badura, A., & Romantsik, O. (2023). Stem cell-based interventions for the treatment of stroke in newborn infants. The Cochrane database of systematic reviews, 11(11), CD015582. https://doi.org/10.1002/14651858.CD015582.pub2	Wrong outcome
Pennington, L., Parker, N. K., Kelly, H., & Miller, N. (2016). Speech therapy for children	Wrong

with dysarthria acquired before three years of age. The Cochrane database of systematic reviews, 7(7), CD006937. https://doi.org/10.1002/14651858.CD006937.pub3	outcome
Guttmann, K., Flibotte, J., & DeMauro, S. B. (2018). Parental Perspectives on Diagnosis and Prognosis of Neonatal Intensive Care Unit Graduates with Cerebral Palsy. The Journal of pediatrics, 203, 156–162. https://doi.org/10.1016/j.jpeds.2018.07.089	Wrong outcome
Reid, S. M., Meehan, E. M., Reddihough, D. S., & Harvey, A. R. (2018). Dyskinetic vs Spastic Cerebral Palsy: A Cross-sectional Study Comparing Functional Profiles, Comorbidities, and Brain Imaging Patterns. Journal of child neurology, 33(9), 593–600. https://doi.org/10.1177/0883073818776175	Wrong outcome
Tantsis, E. M., Mohammad, S. S., Paget, S. P., Virella-Perez, Y. I., Han, V. X., Hadi, D., Goldman, C., Farrar, M. A., Fahey, M., Dale, R. C., & GENE-CP study group (2025). Genetic testing in cerebral palsy with clinical and neuroimaging variables. Developmental medicine and child neurology, 10.1111/dmcn.16323. Advance online publication. https://doi.org/10.1111/dmcn.16323	Wrong outcome
Patel, D. R., Bovid, K. M., Rausch, R., Ergun-Longmire, B., Goetting, M., & Merrick, J. (2024). Cerebral palsy in children: A clinical practice review. Current problems in pediatric and adolescent health care, 54(11), 101673. https://doi.org/10.1016/j.cppeds.2024.101673	Wrong outcome
Maitre, N. L., Byrne, R., Duncan, A., Dusing, S., Gaebler-Spira, D., Rosenbaum, P., Winter, S., CP EDI Consensus Group, & Canadian Neonatal Follow-up Network (2022). "High-risk for cerebral palsy" designation: A clinical consensus statement. Journal of pediatric rehabilitation medicine, 15(1), 165–174. https://doi.org/10.3233/PRM-220030	Wrong outcome
Brun, C., Traverse, É., Granger, É., & Mercier, C. (2021). Somatosensory deficits and neural correlates in cerebral palsy: a scoping review. Developmental medicine and child	Wrong outcome

neurology, 63(12), 1382–1393. https://doi.org/10.1111/dmcn.14963	
Aravamuthan, B. R., Fehlings, D., Shetty, S., Fahey, M., Gilbert, L., Tilton, A., & Kruer, M. C. (2021). Variability in Cerebral Palsy Diagnosis. Pediatrics, 147(2), e2020010066. https://doi.org/10.1542/peds.2020-010066	Wrong outcome
Richards, C. L., & Malouin, F. (2013). Cerebral palsy: definition, assessment and rehabilitation. Handbook of clinical neurology, 111, 183–195. https://doi.org/10.1016/B978-0-444-52891-9.00018-X	Wrong outcome
Phillips, J. P., Sullivan, K. J., Burtner, P. A., Caprihan, A., Provost, B., & Bernitsky-Beddingfield, A. (2007). Ankle dorsiflexion fMRI in children with cerebral palsy undergoing intensive body-weight-supported treadmill training: a pilot study. Developmental medicine and child neurology, 49(1), 39–44. https://doi.org/10.1017/s0012162207000102.x	Wrong population
Boog G. (2010). Asphyxie périnatale et infirmité motrice d'origine cérébrale (I--le diagnostic) [Cerebral palsy and perinatal asphyxia (I--diagnosis)]. Gynecologie, obstetrique & fertilite, 38(4), 261–277. https://doi.org/10.1016/j.gyobfe.2010.02.009	Wrong outcome
Keogh, J. M., & Badawi, N. (2006). The origins of cerebral palsy. Current opinion in neurology, 19(2), 129–134. https://doi.org/10.1097/01.wco.0000218227.35560.0d	Wrong outcome
Cooper, M. S., Fahey, M. C., & Mackay, M. T. (2022). Making waves: The changing tide of cerebral palsy. Journal of paediatrics and child health, 58(11), 1929–1934. https://doi.org/10.1111/jpc.16186	Wrong outcome
Tian, F., Delgado, M. R., Dhamne, S. C., Khan, B., Alexandrakis, G., Romero, M. I., Smith, L., Reid, D., Clegg, N. J., & Liu, H. (2010). Quantification of functional near infrared spectroscopy to assess cortical reorganization in children with cerebral palsy. Optics	Wrong outcome

express, 18(25), 25973–25986. https://doi.org/10.1364/OE.18.025973	
Tian, F., Delgado, M. R., Dhamne, S. C., Khan, B., Alexandrakis, G., Romero, M. I., Smith, L., Reid, D., Clegg, N. J., & Liu, H. (2010). Quantification of functional near infrared spectroscopy to assess cortical reorganization in children with cerebral palsy. Optics express, 18(25), 25973–25986. https://doi.org/10.1364/OE.18.025973	Wrong outcome
Maaoui, R., Karoui, S., Hfaidh, M., Mouhli, N., Ezzine, Z., Ksibi, I., Rahali, H., Barakizou, H., & Hamdi, K. (2023). Cerebral palsy of the child in rehabilitation environment: epidemiologic and clinical profile and therapeutic modalities. La Tunisie medicale, 101(7), 642–647.	Wrong outcome
Maurer U. (2002). Ursachen der Zerebralparese und klassische Behandlungsmöglichkeiten [Etiologies of cerebral palsy and classical treatment possibilities]. Wiener medizinische Wochenschrift (1946), 152(1-2), 14–18. https://doi.org/10.1046/j.1563-258x.2002.01111.x	Wrong outcome
McLellan A. (2008). Epilepsy--an additional risk factor for psychological problems in cerebral palsy. Developmental medicine and child neurology, 50(10), 727. https://doi.org/10.1111/j.1469-8749.2008.03103.x	Wrong outcome
Viswanath, M., Jha, R., Gambhirao, A. D., Kurup, A., Badal, S., Kohli, S., Parappil, P., John, B. M., Adhikari, K. M., Kovilapu, U. B., & Sondhi, V. (2023). Comorbidities in children with cerebral palsy: a single-centre cross-sectional hospital-based study from India. BMJ open, 13(7), e072365. https://doi.org/10.1136/bmjopen-2023-072365	Wrong outcome
Pueyo, R., Junqué, C., & Vendrell, P. (2003). Neuropsychologic differences between bilateral dyskinetic and spastic cerebral palsy. Journal of child neurology, 18(12), 845–850. https://doi.org/10.1177/08830738030180120 4	Wrong outcome
Ozturk, A. T., Berk, A. T., & Yaman, A. (2013). Ocular disorders in children with spastic	Wrong

subtype of cerebral palsy. International journal of ophthalmology, 6(2), 204–210. https://doi.org/10.3980/j.issn.2222-3959.2013.02.19	outcome
Bacciu, A., Pasanisi, E., Vincenti, V., Ormitti, F., Di Lella, F., Guida, M., Berghenti, M., & Bacciu, S. (2009). Cochlear implantation in children with cerebral palsy. A preliminary report. International journal of pediatric otorhinolaryngology, 73(5), 717–721. https://doi.org/10.1016/j.ijporl.2009.01.010	Wrong outcome
Pilato, F., Dileone, M., Capone, F., Profice, P., Caulo, M., Battaglia, D., Ranieri, F., Oliviero, A., Florio, L., Graziano, A., Di Rocco, C., Massimi, L., & Di Lazzaro, V. (2009). Unaffected motor cortex remodeling after hemispherectomy in an epileptic cerebral palsy patient. A TMS and fMRI study. Epilepsy research, 85(2-3), 243–251. https://doi.org/10.1016/j.eplepsyres.2009.03.016	Wrong outcome
Harat, M., Radziszewski, K., Rudaś, M., Okoń, M., & Galanda, M. (2009). Clinical evaluation of deep cerebellar stimulation for spasticity in patients with cerebral palsy. Neurologia i neurochirurgia polska, 43(1), 36–44.	Wrong outcome
Pueyo, R., Junqué, C., Vendrell, P., Narberhaus, A., & Segarra, D. (2009). Neuropsychologic impairment in bilateral cerebral palsy. Pediatric neurology, 40(1), 19–26. https://doi.org/10.1016/j.pediatrneurol.2008.08.003	Wrong outcome
Hoffman, R. M., Trevarrow, M. P., Lew, B. J., Wilson, T. W., & Kurz, M. J. (2024). Alpha oscillations during visual selective attention are aberrant in youth and adults with cerebral palsy. Cerebral cortex (New York, N.Y. : 1991), 34(9), bhae365. https://doi.org/10.1093/cercor/bhae365	Wrong outcome
Kukka, A.J., Bhattarai, P., Sundelin, H.E.K. et al. 'We did everything by phone': a qualitative study of mothers' experience of smartphone-aided screening of cerebral palsy in	Wrong outcome

Kathmandu, Nepal. BMC Pediatr 24, 357 (2024). https://doi.org/10.1186/s12887-024-04829-5	
Cooper, M. S., Mackay, M. T., Dagia, C., Fahey, M. C., Howell, K. B., Reddihough, D., Reid, S., & Harvey, A. S. (2023). Epilepsy syndromes in cerebral palsy: varied, evolving and mostly self-limited. Brain: a journal of neurology, 146(2), 587–599. https://doi.org/10.1093/brain/awac274	Wrong outcome
Brown, A., Tornberg, Å. B., & Kristensson Hallström, I. (2024). Parents' lived experience of early risk assessment for cerebral palsy in their young child using a mobile application after discharge from hospital in the newborn period. Annals of medicine, 56(1), 2309606. https://doi.org/10.1080/07853890.2024.2309606	Wrong outcome
Kleeren, L., Mailleux, L., McLean, B., Elliott, C., Dequeker, G., Van Campenhout, A., de Xivry, J. O., Verheyden, G., Ortibus, E., Klingels, K., & Feys, H. (2024). Does somatosensory discrimination therapy alter sensorimotor upper limb function differently compared to motor therapy in children and adolescents with unilateral cerebral palsy: study protocol for a randomized controlled trial. Trials, 25(1), 147. https://doi.org/10.1186/s13063-024-07967-4	Wrong outcome
Chen, Y. H., Chen, C. L., Hong, W. H., Chen, C. Y., Chung, C. Y., Wu, K. P. H., Wu, C. Y., & Lin, K. C. (2024). Precision in Progress: Unraveling the Clinimetric Properties of Beery-Buktenica Developmental Test of Visual-Motor Integration in Children With Cerebral Palsy Across Diverse Motor Severities. Pediatric neurology, 161, 139–143. https://doi.org/10.1016/j.pediatrneurol.2024.09.017	Wrong outcome
Boyd, R. N., Novak, I., Morgan, C., Bora, S., Sakzewski, L., Ware, R. S., Comans, T., Fahey, M. C., Whittingham, K., Trost, S., Pannek, K., Pagnozzi, A., Mcintyre, S., Badawi, N., Smithers Sheedy, H., Palmer, K. R., Burgess, A., Keramat, A., Bell, K., Hines, A., … Oftedal, S. (2023). School readiness of children at high risk of cerebral palsy randomised to early	Wrong outcome

neuroprotection and neurorehabilitation: protocol for a follow-up study of participants from four randomised clinical trials. BMJ open, 13(2), e068675. https://doi.org/10.1136/bmjopen-2022-068675	
Shevell, A. H., & Shevell, M. (2013). Doing the "talk": disclosure of a diagnosis of cerebral palsy. Journal of child neurology, 28(2), 230–235. https://doi.org/10.1177/0883073812471430	Wrong outcome
Zhao, J., Qiu, Y., & Wang, H. (2025). Nutritional risk screening and nutritional assessment for children with cerebral palsy: A review of the current research status and future directions. Clinical nutrition ESPEN, 65, 382–389. https://doi.org/10.1016/j.clnesp.2024.12.018	Wrong outcome
Surveillance of Cerebral Palsy in Europe (2000). Surveillance of cerebral palsy in Europe: a collaboration of cerebral palsy surveys and registers. Surveillance of Cerebral Palsy in Europe (SCPE). Developmental medicine and child neurology, 42(12), 816–824. https://doi.org/10.1017/s0012162200001511	Wrong outcome
Fabricius, R. A., Larsen, M. L., Debes, N. M., Rackauskaite, G., & Hoei-Hansen, C. E. (2024). Impact of a National Follow-Up Program on the Age at Diagnosis for Cerebral Palsy. Pediatric neurology, 152, 56–61. https://doi.org/10.1016/j.pediatrneurol.2023.11.008	Wrong outcome
Maitre, N. L., Burton, V. J., Duncan, A. F., Iyer, S., Ostrander, B., Winter, S., Ayala, L., Burkhardt, S., Gerner, G., Getachew, R., Jiang, K., Lesher, L., Perez, C. M., Moore-Clingenpeel, M., Lam, R., Lewandowski, D. J., & Byrne, R. (2020). Network Implementation of Guideline for Early Detection Decreases Age at Cerebral Palsy Diagnosis. Pediatrics, 145(5), e20192126. https://doi.org/10.1542/peds.2019-2126	Wrong outcome
Jaleel, F., Rust, A., Cheung, S., Pearson, T. S., Ueda, K., Robichaux-Viehoever, A., Leger, K., Chintalapati, K., Guez-Barber, D., Shusterman, M., & Aravamuthan, B. (2024). Caregiver	Wrong outcome

descriptions of dystonia in cerebral palsy. Annals of clinical and translational neurology, 11(2), 242–250. https://doi.org/10.1002/acn3.51941	
Morgan, C., Badawi, N., Boyd, R. N., Spittle, A. J., Dale, R. C., Kirby, A., Hunt, R. W., Whittingham, K., Pannek, K., Morton, R. L., Tarnow-Mordi, W., Fahey, M. C., Walker, K., Prelog, K., Elliott, C., Valentine, J., Guzzetta, A., Olivey, S., GAME study team, & Novak, I. (2023). Harnessing neuroplasticity to improve motor performance in infants with cerebral palsy: a study protocol for the GAME randomised controlled trial. BMJ open, 13(3), e070649. https://doi.org/10.1136/bmjopen-2022-070649	Wrong outcome
Özdemir, F. M. A., Taşçi Yildiz, Y., Gümüşer Cinni, R., Zenciroğlu, A., & Yüksel, D. (2024). Short- and long-term neurological outcomes of congenital cytomegalovirus infection. Turkish journal of medical sciences, 54(3), 529–536. https://doi.org/10.55730/1300-0144.5819	Wrong outcome
Santana Almansa, A., Gable, D. L., Frazier, Z., Sveden, A., Quinlan, A., Chopra, M., Lewis, S. A., Kruer, M., Poduri, A., & Srivastava, S. (2024). Clinical utility of a genetic diagnosis in individuals with cerebral palsy and related motor disorders. Annals of clinical and translational neurology, 11(2), 251–262. https://doi.org/10.1002/acn3.51942	Wrong outcome
Eliasson, A. C., Nordstrand, L., Ek, L., Lennartsson, F., Sjöstrand, L., Tedroff, K., & Krumlinde-Sundholm, L. (2018). The effectiveness of Baby-CIMT in infants younger than 12 months with clinical signs of unilateral-cerebral palsy; an explorative study with randomized design. Research in developmental disabilities, 72, 191–201. https://doi.org/10.1016/j.ridd.2017.11.006	Wrong outcome
Parikh N. A. (2018). Are Structural Magnetic Resonance Imaging and General Movements Assessment Sufficient for Early, Accurate Diagnosis of Cerebral Palsy?. JAMA pediatrics,	Wrong outcome

172(2), 198–199. https://doi.org/10.1001/jamapediatrics.2017.4812	
Pham, R., Mol, B. W., Gecz, J., MacLennan, A. H., MacLennan, S. C., Corbett, M. A., van Eyk, C. L., Webber, D. L., Palmer, L. J., & Berry, J. G. (2020). Definition and diagnosis of cerebral palsy in genetic studies: a systematic review. Developmental medicine and child neurology, 62(9), 1024–1030. https://doi.org/10.1111/dmcn.14585	Wrong outcome
Dan B. (2020). How useful is the diagnosis of ataxic cerebral palsy?. Developmental medicine and child neurology, 62(3), 264. https://doi.org/10.1111/dmcn.14453	Wrong outcome
McNamara, L., Scott, K. M., Boyd, R. N., & Novak, I. (2021). Consensus of physician behaviours to target for early diagnosis of cerebral palsy: A Delphi study. Journal of paediatrics and child health, 57(7), 1009–1015. https://doi.org/10.1111/jpc.15369	Wrong outcome
Hoei-Hansen, C. E., Weber, L., Johansen, M., Fabricius, R., Hansen, J. K., Viuff, A. F., Rønde, G., Hahn, G. H., Østergaard, E., Duno, M., Larsen, V. A., Madsen, C. G., Røhder, K., Elvrum, A. G., Laugesen, B., Ganz, M., Madsen, K. S., Willerslev-Olsen, M., Debes, N. M., Christensen, J., … Rackauskaite, G. (2023). Cerebral Palsy - Early Diagnosis and Intervention Trial: protocol for the prospective multicentre CP-EDIT study with focus on diagnosis, prognostic factors, and intervention. BMC pediatrics, 23(1), 544. https://doi.org/10.1186/s12887-023-04312-7	Wrong outcome
Te Velde, A., Tantsis, E., Novak, I., Badawi, N., Berry, J., Golland, P., Korkalainen, J., McMurdo, R., Shehata, R., & Morgan, C. (2021). Age of Diagnosis, Fidelity and Acceptability of an Early Diagnosis Clinic for Cerebral Palsy: A Single Site Implementation Study. Brain sciences, 11(8), 1074. https://doi.org/10.3390/brainsci11081074	Wrong outcome
